# Bayesian Optimization for Multicomponent Supramolecular Systems

**DOI:** 10.1021/jacs.5c08539

**Published:** 2025-09-04

**Authors:** Stef A. H. Jansen, Albert J. Markvoort, Freek V. de Graaf, Martin G.T.A. Rutten, Patricia Y. W. Dankers, Ghislaine Vantomme, Tom F. A. de Greef, E. W. Meijer

**Affiliations:** †Institute for Complex Molecular Systems, ‡Laboratory of Macromolecular and Organic Chemistry, §Synthetic Biology Group, ∥Laboratory of Chemical Biology, 201226 Eindhoven University of Technology, 5600 MB Eindhoven, The Netherlands; ⊥ Institute for Molecules and Materials, Radboud University, 6500 GL Nijmegen, The Netherlands; # Center for Living Technologies, Eindhoven-Wageningen-Utrecht Alliance, 3584 CS Utrecht, The Netherlands; ∇ School of Chemistry and RNA Institute, University of New South Wales, 2052 Sydney, Australia; ○ Max Planck Institute for Polymer Research, Ackermannweg 10, 55128 Mainz, Germany

## Abstract

The diversity of noncovalent interactions makes the design space of multicomponent molecular systems highly complex. To efficiently explore supramolecular design space, data-driven strategies are needed. Here, we demonstrate a methodological framework for the targeted design of multicomponent molecular systems with noncovalent interactions using Bayesian optimization. Its effective applicability to supramolecular polymers is illustrated by three representative cases that reveal accelerated exploration of diverse multicomponent systems with a universal Bayesian optimization framework. The number of experiments required to arrive at optimal compositions is significantly reduced compared to random or uninformed sampling strategies, enabling the experimental study of high-dimensional design spaces. In this way, we can tune the formulation of intricate mixtures and achieve tailored macroscopic properties with minimal experimental effort. Our results show that Bayesian optimization is a general tool for developing multicomponent supramolecular systems with designed functionality.

## Graphical abstract

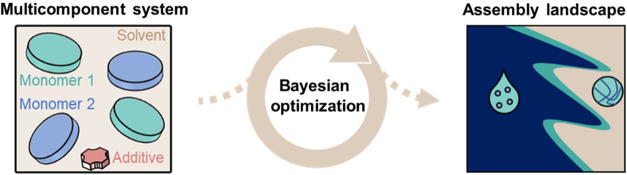

## Introduction

In supramolecular systems, molecular components assemble synchronously through noncovalent interactions, in contrast to the sequential formation of covalent bonds in organic synthesis.^1,2^ Assemblies that consist of multiple components can achieve properties that are unattainable with single components, making coassembly an attractive approach to obtaining functional molecular systems.^3,4^ Nevertheless, the functionality of multicomponent self-assembled systems is optimal within a narrow range of compositions and conditions (Figure 1a).^5,6^ The vast supramolecular design space resulting from the diversity of noncovalent interactions makes the discovery of optimal formulations challenging.^7^

**Figure 1:**
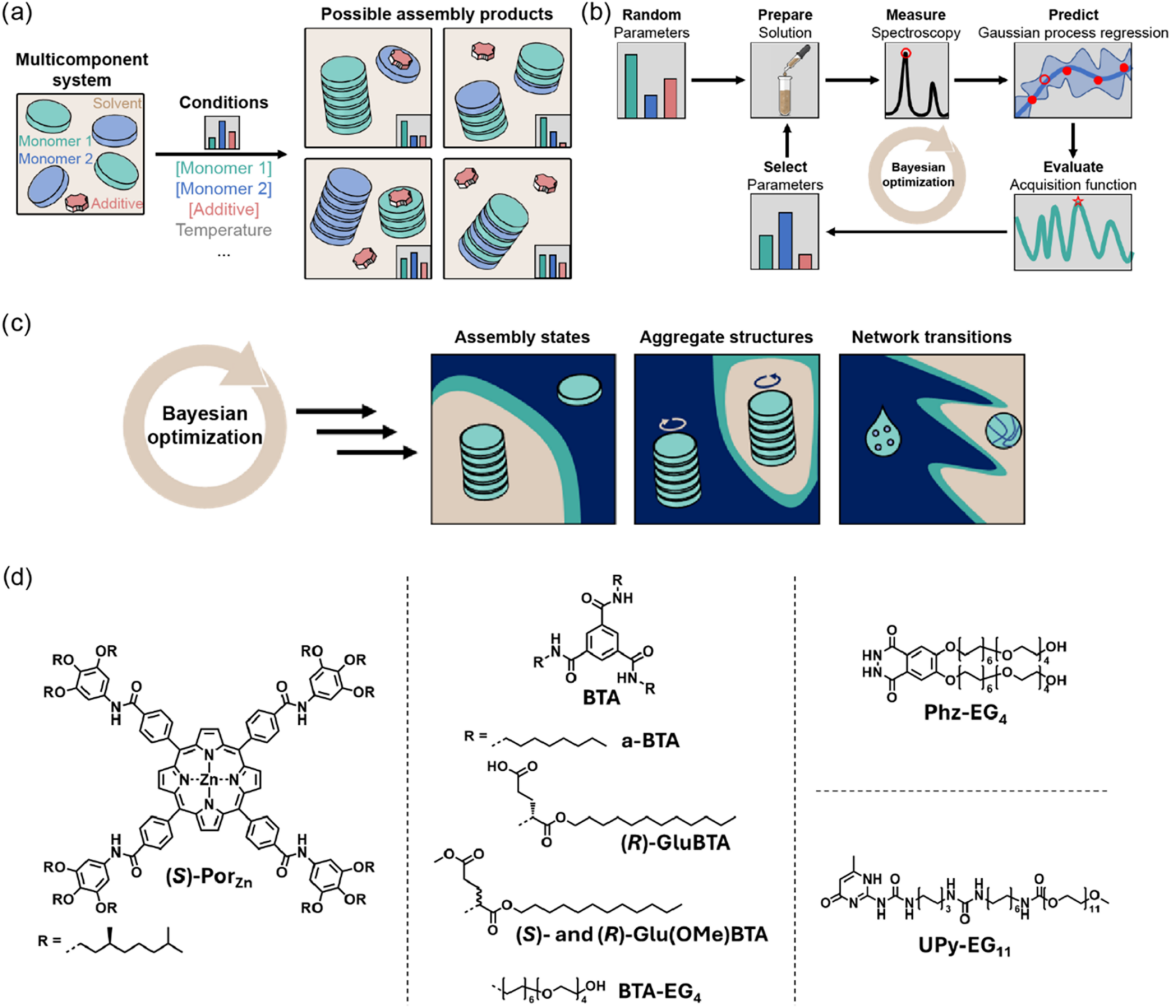
Bayesian optimization applied to multicomponent supramolecular systems. (a) Multicomponent supramolecular systems form different assembly products, depending on the composition and conditions. In this example, two monomers assemble into different assembly products in the presence of solvent and an additive. (b) Schematic overview of Bayesian optimization workflow applied to supramolecular design spaces in order to efficiently explore supramolecular design space. Random parameters are initially selected, after which iterations are performed of (i) solution preparation according to the selected parameters, (ii) UV/vis or circular dichroism spectroscopic measurements of the solution, (iii) Gaussian process regression based on the spectroscopic data, (iv) explorative, exploitative or hybrid acquisition function evaluation to (v) select most informative new parameters. These steps can be continuously iterated until the prediction from Gaussian process regression is satisfactory. (c) Bayesian optimization is applied to map the phase diagrams of three different multicomponent supramolecular systems, allowing the accelerated discovery of (i) assembly states, (ii) aggregate structures, and (iii) network transitions. (d) Molecular structures of the monomer library (containing **Por**-, **BTA**-, **Phz**- and **UPy**-based monomers) studied in this report.

Currently, the most effective approach for exploring supramolecular design spaces utilizes global analyses with mechanistic models.^8,9^ However, as the number of monomers, additives, and solvent interactions increases, the derivation of these models becomes increasingly difficult due to the presence of unknown assembly pathways.^10,11^ In addition, the large number of model parameters prevents their accurate estimation using nonlinear least-squares methods.^12^ Therefore, an unbiased and universal approach based on experimental data is desired to efficiently design multicomponent supramolecular systems. In these highly dimensional parameter spaces, random experimental sampling is laborious and inefficient and often does not lead to the identification of optimal formulations.^13^ Thus, efficient methods need to be developed that can guide design space exploration to achieve tailored functional molecular systems.

Bayesian optimization has emerged as a powerful computational tool that enables systematic exploration of chemical parameter spaces in a low-data regime, thereby accelerating the discovery of optimal chemical systems (Figure 1b).^14,15^ Bayesian optimization has already facilitated impressive achievements in molecular design,^16^ material design,^17–20^ and reaction optimization.^21–23^ Inspired by these pioneering studies, here we translate this method to the supramolecular design space as illustrated by three representative case studies (Figure 1c). First, we validate Bayesian optimization for accelerated exploration of assembly landscapes,^24^ showing how Bayesian optimization can efficiently map complex phase boundaries. Next, we demonstrate how the same Bayesian optimization framework can be applied to locate optimal parameter values in supramolecular design space for a specific target property—in this case, maximizing the helicity change upon covalent modification of supramolecular copolymers.^25^ Finally, we will illustrate its potential for guiding the design of tailored multicomponent supramolecular materials by exploiting Bayesian optimization to map the interactions between supramolecular polymers and surfactants in water.^26^ The resulting insights enabled the formulation of a four-component system that exhibits reentrant transitions in the supramolecular network upon dilution. Overall, our study showcases the potential of Bayesian optimization to accelerate the targeted design of functional supramolecular systems, which drives innovation in nanotechnology.

## Results and Discussion

### Mapping Assembly Landscapes by Bayesian Optimization

We have recently reported a methodology to map assembly landscapes of supramolecular polymer systems (e.g., **(** ***S*** **)-Por**_**Zn**_ with 4280 equiv of ethanol in methylcyclohexane, Figure 1d) using thermodynamic mass-balance models.^24^ In the current study, we leveraged this model to efficiently simulate measurements, enabling rapid testing and evaluation of Bayesian optimization (BO) strategies without the need for time-consuming experiments. To explore the mapping of assembly landscapes using BO (Figure 2a), we integrated active learning techniques^27^ into a straightforward framework with different acquisition functions and applied it to the model assembly landscape of **(** ***S*** **)-Por**_**Zn**_ in methylcyclohexane (MCH) with ethanol as additive, where the temperature and concentration were varied. The selected BO framework iteratively uses (1) Gaussian process regression (GPR) to make a prediction of the assembly landscape based on the data points already sampled, (2) minimization of an acquisition function to select the most informative new data point(s) to collect, (3) simulation of the selected data point(s) using the mass-balance model. Explorative (favoring uncertain regions), exploitative (favoring regions predicted to yield the target of interest), and hybrid (balancing exploration and exploitation) acquisition functions were applied to target the monomer–polymer transition. The performance was evaluated by tracking the *R* ^2^ of the GPR predictions at each iteration (Figure 2b) with respect to the model ground truth (Figure 2c). The BO framework outperformed random sampling irrespective of the acquisition function used, and a hybrid acquisition function showed the best performance, reducing the number of experiments to reach an *R* ^2^ above 0.9 by approximately 50% compared to random sampling (Figures 2b and S8). Moreover, the hybrid acquisition function (ε = 0.5) appeared to be the most robust in the optimization (Table S1).

**Figure 2:**
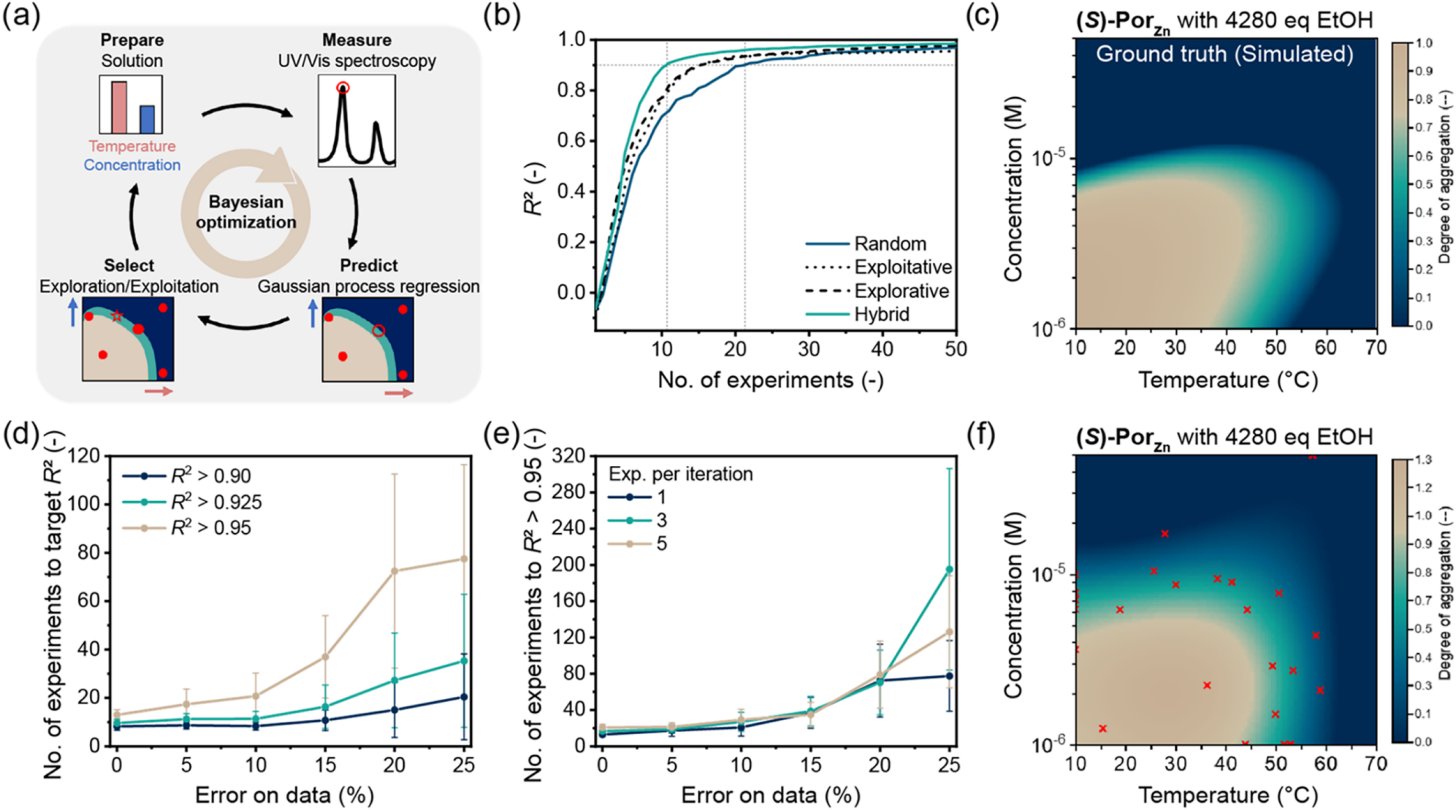
Accelerated mapping of assembly landscapes with Bayesian optimization. (a) Overview of BO workflow to investigate assembly states as a function of temperature and concentration. (b) *R* ^2^ of Gaussian process surrogate model prediction compared to the ground truth in (c) during BO, with different sample selection methods (random sampling or explorative, exploitative or hybrid ε = 0.5 acquisition functions). Average of 100 optimizations with simulated data, starting with four random data points. (c) Ground truth of assembly landscape of **(** ***S*** **)-Por**_**Zn**_ with 4280 equiv ethanol in methylcyclohexane, simulated with a thermodynamic mass-balance model. (d) Number of experiments required to obtain different posterior mean accuracies as a function of the experimental error. Average of 100 optimizations with simulated data points, starting with four random data points. Error bars indicate 0.5σ. (e) Number of experiments required to obtain a prediction with an *R* ^2^ above 0.95 as a function of the experimental error for different batch sizes per iteration. Average of 100 optimizations with simulated data points, starting with four random data points. Error bars indicate 0.5σ. (f) Posterior mean or predicted assembly landscape after 25 experiments of BO with the hybrid acquisition function, starting with four random experimental data points and measuring three data points per iteration. Red crosses indicate the experimentally measured data points.

To assess the robustness of BO, we simulated artificial data inaccuracies and monitored the effect on BO performance using simulations with the mass-balance model. We observed that data accuracy becomes increasingly important as a more accurate prediction is targeted (Figure 2d). In this case, the nonlinearity suggests that improving data accuracy should be prioritized especially when the experiments are costly. To accelerate BO in cases with time-consuming data point evaluation, multiple samples can be selected and acquired in parallel in each iteration. The batch sizes were chosen for practical reasons, considering the number of parallel experiments that the instrument can perform and the rough estimate on the total number of required experiments. The most suitable batch size should be reconsidered for experimental applications to different systems. When the data inaccuracy is less than 20%, sampling 3 or 5 data points per iteration appears to have little impact on the optimization performance (Figure 2e). When the experimental error is higher, small experimental batches should be used to minimize the number of required experiments.

To validate this approach experimentally, we used UV/vis spectroscopy to determine the degree of aggregation and map this assembly landscape with BO–Hybrid. The model successfully selected data points for new experiments at the targeted model–polymer transition and after 28 experiments, the constructed assembly landscape (Figure 2f) already showed major similarities with the assembly landscape predicted by the mass-balance model (*R* ^2^ = 0.86).

Together, these results validate the use of BO as an experimental approach for the accelerated exploration of assembly landscapes of multicomponent supramolecular systems and provide guidelines for the efficient use of BO. As a next step, the same BO framework can be extended to locate optimum conditions for user-defined target properties in a supramolecular design space, as demonstrated in the following optimization case study.

### Bayesian Optimization of Covalent Modification in Supramolecular Copolymers

In addition to the efficient exploration of assembly landscapes, multicomponent supramolecular systems require condition optimization to achieve the targeted properties from the assembled structures. We aimed to validate the presented BO framework for optimization in the chemical conversion of a chiral supramolecular system consisting of three coassembling benzene-1,3,5-tricarboxamides (BTA) monomers (Figures 1d and 3a). The covalent modification of the chiral glutamic acid BTA (**Glu-BTA**) to a glutamic acid 5-methyl ester BTA (**Glu­(OMe)-BTA**) under assembly conditions (50 μM in MCH) has been used to alter the copolymer helicity (Figure 3b).^25^ We aimed to enhance this helicity change (characterized by the change in circular dichroism signal, ΔCD) by applying BO to the formulation of the initial BTA mixture, varying the ratios of the three comonomers.

**Figure 3:**
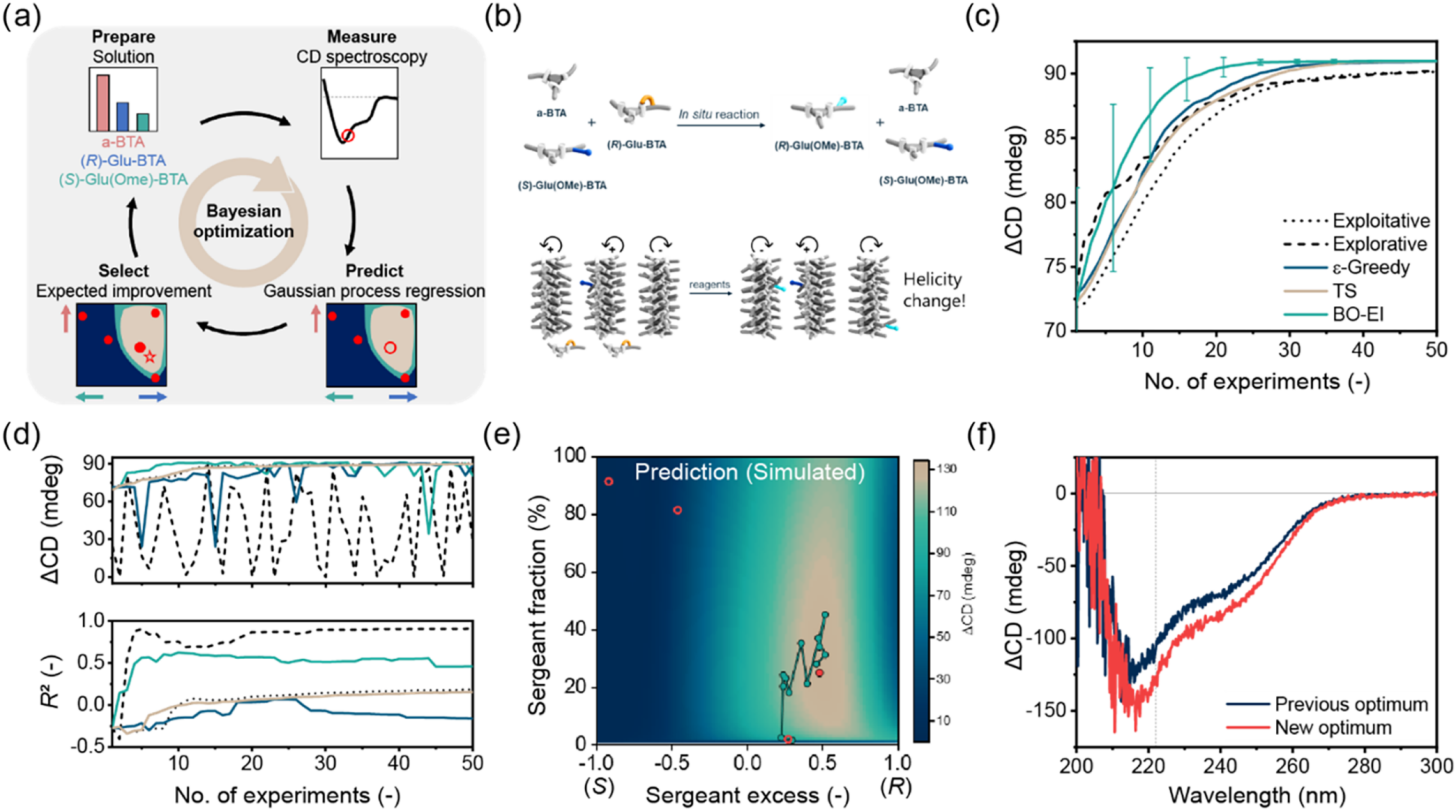
Bayesian optimization for covalent modification of supramolecular polymer systems. (a) Overview of BO workflow to investigate aggregate structures as a function of the comonomer ratios. (b) Schematic representation of *in situ* reaction on **(** ***R*** **)-Glu-BTA** to change the helicity of copolymers in a diluted Majority-rules system with **a-BTA** and **(** ***S*** **)-Glu­(OMe)-BTA**. (c) ΔCD as a measure for the helicity change upon covalent modification, monitored during BO with expected improvement acquisition function (BO-EI), compared to different sample selection methods: exploitative and explorative selection, ε-Greedy randomization probability 0.1, and Thompson sampling (TS). Average of 50 optimizations with simulated data, starting with three random data points. Error bars indicate 0.5σ. (d) ΔCD (top) and *R* ^2^ (bottom) of the data points selected by the optimizers in c during a single optimization, starting with the same initial data set. (e) Simulated ΔCD as a function of sergeant excess and sergeant fraction, using a thermodynamic mass-balance model. The randomly selected initial data set is indicated by the open red circles. The first 13 steps of BO-EI from d are shown in green. The optimum found after BO-EI is marked in red. (f) Experimental ΔCD spectra of the optimum found by BO-EI, compared to the observed maximum in the previous report.^28^.

For this optimization, we implemented the Expected Improvement acquisition function^29^ in the established BO framework to target the maximum change in copolymer helicity. For data simulation, we extended the two-component copolymerization mass-balance model^30^ to three components. By performing 50 runs of BO for each acquisition function with different random initial data points, we found that the EI acquisition function consistently outperformed purely explorative sampling, which serves as a systematic and nontargeted baseline. Furthermore, EI was more efficient than purely exploitative sampling and two other sampling methods that balance exploration and exploitation (i.e., ε-Greedy and Thompson sampling, Figures 3c and S10), showing significantly more robust optimization of simulated ΔCD in 50 BO iterations (Table S2). When tracking the selected data points during optimization, all acquisition functions (except the explorative function) successfully selected samples with large helicity changes, but the EI function simultaneously explored the entire parameter space more efficiently (Figure 3d,e). BO-EI generally approached the optimum at 24.8 mol % sergeant with 0.48 (***R***)-sergeant excess in around 25 simulated experiments, depending on the initial data set.

To experimentally verify the optimal system composition found with BO and simulated data, we used CD spectroscopy to experimentally measure the sum of copolymer helicities (Figure 3f). For this purpose, we measured the CD signal of the mixture before covalent modification (**a-BTA**+**(** ***R*** **)-Glu-BTA**+**(** ***S*** **)-Glu­(OMe)-BTA**), and of the mixture of components that would be obtained after 100% conversion of **(** ***R*** **)-Glu-BTA** (**a-BTA**+**(** ***R*** **)-Glu­(OMe)-BTA**+**(** ***S*** **)-Glu­(OMe)-BTA**). The results indicate a 20% larger helicity change (ΔCD) than observed in experiments in which BO was not employed.^25^ Collectively, these results further validate that BO can accelerate the optimization of multicomponent supramolecular systems. Importantly, BO can be applied to supramolecular systems that cannot be quantitatively modeled with conventional methods due to their high dimensionality or the intricacy of noncovalent interactions.

### Bayesian Optimization to Tailor Supramolecular Networks in Water

Having established guidelines for the effective use of BO in supramolecular systems, we next sought to elucidate the concentration-dependent interactions between common water-soluble monomers and surfactants that cause hydrogel–solution-hydrogel–solution transitions upon dilution with water.^26^ For this purpose, we applied BO to map the hydrogel–solution transition of the orthogonal **UPy-EG**_**11**_ and **BTA-EG**_**4**_ supramolecular networks individually in the presence of **OTAB** (Figures 1d and 4a,b). We used UV/vis spectroscopy to determine the equivalent monomer concentration in polymers and compared this with the absorption data at the gelation concentration of the respective monomers. We assume that the absorption is correlated with the supramolecular network density and that the network density determines the viscosity of the solution. This provided a simplified estimation of the phase (i.e., solution or hydrogel) with basic UV/vis spectroscopic experiments, which accelerated the experimental BO trials. The assembly landscapes obtained with BO were plotted as a function of **OTAB** concentration and monomer equivalents with respect to **OTAB** (Figures S14 and S15). As a result, the correct amount of each monomer with respect to **OTAB** can be determined from the combined assembly landscapes to achieve the supramolecular network transitions sequentially in Milli-Q (MQ)-H_2_O (Figure 4d). The observed hydrogel–solution boundary from **UPy-EG**_**11**_ in the presence of **OTAB** (red trace in Figures 4d and S14) closely followed the gelation concentration of **UPy-EG**_**11**_ (∼27.2 mM), indicating a minimal effect of **OTAB** on **UPy-EG**_**11**_ assemblies. In contrast, **BTA-EG**_**4**_ showed a preferential interaction with **OTAB** at higher concentrations, resulting in solution-hydrogel–solution transitions upon dilution for a minimum of 0.13 mol equiv of **BTA-EG**_**4**_ to **OTAB** (blue trace in Figures 4d and S15). These results support and explain that orthogonal supramolecular networks can exhibit dilution-induced hydrogel–solution-hydrogel–solution transitions as reported for the **OTAB:UPy-EG**_**11**_:**BTA-EG**_**4**_ system (1:0.047:0.151 molar ratio).

**Figure 4:**
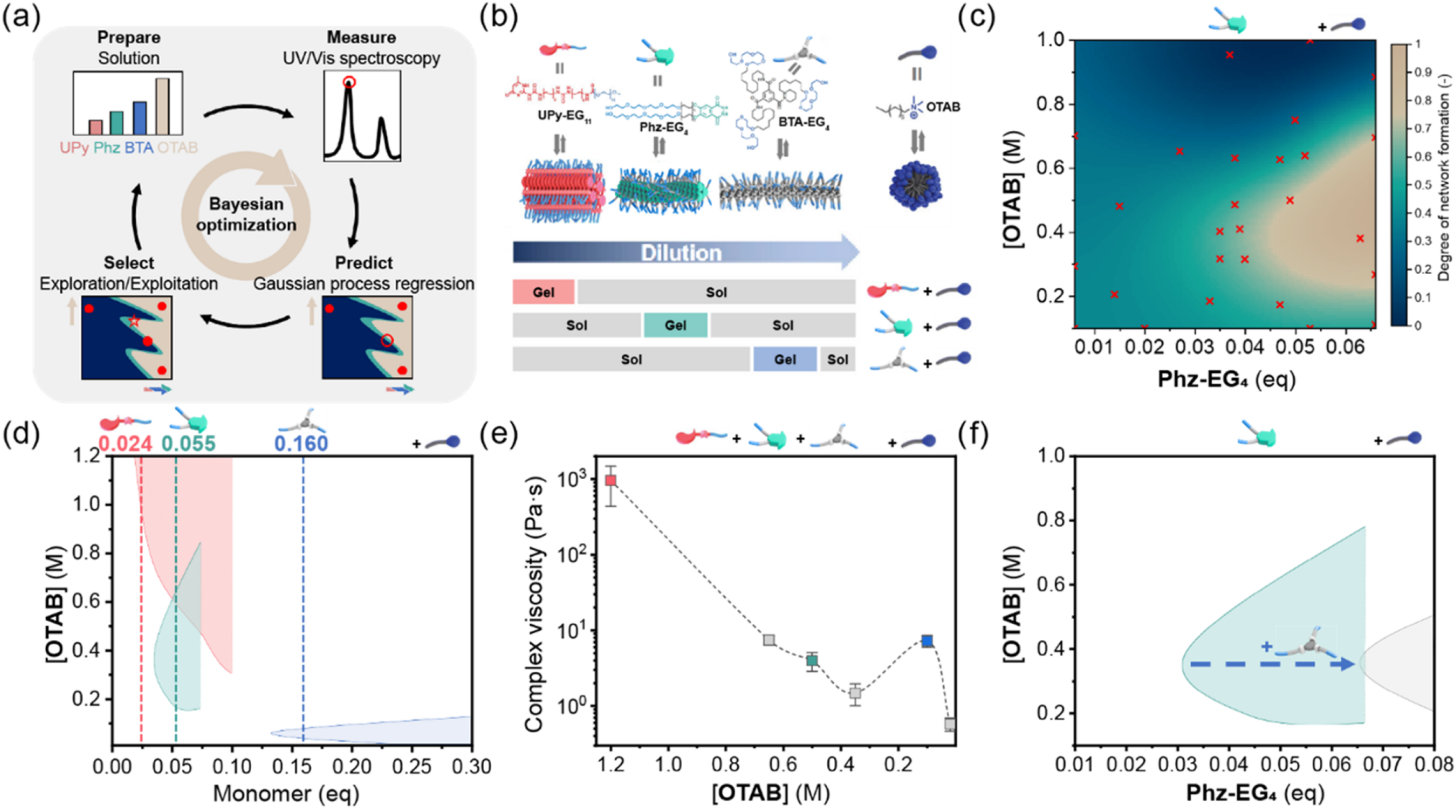
Bayesian optimization to tailor network transitions of multicomponent supramolecular polymer systems. (a) Overview of BO workflow to investigate supramolecular network transitions. The concentrations of four components were optimized with the BO framework to obtain re-entrant viscosity transitions, as characterized with UV/vis spectroscopy. (b) Chemical structures and schematic representation of multicomponent supramolecular system of **UPy-EG**_**11**_, **Phz-EG**_**4**_, and **BTA-EG**_**4**_ monomers with **OTAB** (top). Strategy to obtain reentrant viscosity transitions, utilizing orthogonal supramolecular networks that assemble at different concentrations (bottom). (c) Posterior mean or predicted phase diagram of **Phz-EG**_**4**_ with **OTAB** in water, after 27 experiments selected with BO, selecting new one sample per iteration. Red crosses indicate the experimentally measured data points. (d) Combined predicted phase diagrams of individual monomers (**UPy-EG**_**11**_ in red, **Phz-EG**_**4**_ in green and **BTA-EG**_**4**_ in blue) with **OTAB** in water, where the gel phase of the networks is indicated with their respective colors. Selected equivalents of each monomer compared to **OTAB** are indicated with dashed lines to facilitate transitions between three sequential supramolecular networks upon dilution. (e) Complex viscosity points (ω = 1 rad/s, γ = 1%, 20 °C) corresponding to the supramolecular network transitions in the **OTAB**:**UPy-EG**_**11**_:**Phz-EG**_**4**_:**BTA-EG**_**4**_ system (1:0.024:0.055:0.160), with the dashed line to guide the eye. Error bars indicate σ. (f) Combined predicted phase diagram of **Phz-EG**_**4**_ with **OTAB** in water with (gray) and without (green) 0.19 equiv **BTA-EG**_**4**_ with respect to **OTAB**, after 24 and 27 experiments selected with BO, respectively, showing that the assemblies of **Phz-EG**_**4**_ are destabilized by **BTA-EG**_**4**_.

We were next interested in enriching the concentration-dependent mechanical properties further by introducing a fourth component for an additional phase transition, and **Phz-EG**_**4**_ was identified as a potential candidate due to its distinct assembly properties from **UPy-EG**_**11**_ and **BTA-EG**_**4**_ (Figure 4b).^28^ Obtaining one more reentrant transition of the supramolecular networks requires careful tuning of the system composition to avoid overlapping gelation concentrations. The standard methods in the field would require extensive resource-intensive experimental trials, but with BO we obtained the assembly landscape of **Phz-EG**_**4**_ in the presence of **OTAB** in 27 consecutive UV/vis experiments (Figure 4c). The combined assembly landscapes indicate that an **OTAB**:**UPy-EG**_**11**_:**Phz-EG**_**4**_:**BTA-EG**_**4**_ molar ratio of 1:0.024:0.055:0.160 would exhibit three sequential hydrogel–solution transitions upon dilution from 1200 mM to 20 mM **OTAB** with MQ-H_2_O. Approximately 0.5 mL of the sample was prepared in a vial and formed an opaque, self-standing gel at starting concentrations of 1200:28.8:66:192 mM (**OTAB**:**UPy-EG**_**11**_:**Phz-EG**_**4**_:**BTA-EG**_**4**_) with a storage modulus (*G*′) of ∼ 600 Pa and a complex viscosity (η*) of ∼1 kPa·s (Figures 4e and S19). A dilution with MQ-H_2_O to an **OTAB** concentration of 650 mM destabilized the gel and resulted in a viscous suspension with a η* of ∼8 Pa·s (Figures 4e and S20). Since UV/vis spectroscopy of **Phz-EG**_**4**_ with **OTAB** indicated that **Phz-EG**_**4**_ did not aggregate at this concentration due to interaction with **OTAB**, the observed viscosity may be caused by limited solubility of **Phz-EG**_**4**_ in MQ-H_2_O. Unexpectedly, further dilution of the four-component mixture to 500 mM **OTAB** did not result in a self-standing gel supported by a **Phz-EG**_**4**_ network, in contrast to the two-component mixture of **Phz-EG**_**4**_ and **OTAB**. However, we observed a deflection in the complex viscosity by rheological experiments, indicating an increased network formation of **Phz-EG**_**4**_ as was expected from the **Phz-EG**_**4**_:**OTAB** phase diagram. Control experiments confirmed that the absence of a gel was due to nonorthogonality of **Phz-EG**_**4**_ and **BTA-EG**_**4**_, as the phase transition of **Phz-EG**_**4**_ shifted from >0.31 to >0.65 equiv in the presence of **BTA-EG**_**4**_ (Figures 4f and S16). Further dilution to 350 mM **OTAB** yielded a solution (η* ∼ 1 Pa·s) until a transparent, self-standing gel was obtained at 100 mM **OTAB** (η* ∼ 10 Pa·s), consistent with the assembly landscape of **BTA-EG**_**4**_ with **OTAB**. Thus, the **BTA-EG**_**4**_ network appeared to be unaffected by the **BTA-EG**_**4**_:**Phz-EG**_**4**_ interaction, in contrast to the **Phz-EG**_**4**_ network at higher concentrations where **BTA-EG**_**4**_ is present in excess. The **BTA-EG**_**4**_ gel was disrupted after dilution to 20 mM **OTAB**, resulting in a transparent solution. These results demonstrate the successful optimization of gelation concentrations to obtain sequential transitions, while highlighting the importance of considering factors such as solubility and nonorthogonality in the design of multicomponent supramolecular systems. In this case, the interaction between **Phz-EG**_**4**_ and **BTA-EG**_**4**_ likely arises from partial coassembly or competitive binding but further mechanistic studies would be needed to resolve the exact origin. In this case, BO helps flag such emergent behavior, illustrating how this method can reveal nontrivial design constraints that may otherwise be overlooked. Nonetheless, the formulation of this delicate mixture was achieved after only 67 quick and inexpensive spectroscopic experiments. Thus, BO proved to be instrumental in navigating the complexities of multicomponent hydrogels and ultimately optimizing their formulation in a resource-efficient manner.

## Outlook and Conclusions

Following its success in organic chemistry, Bayesian optimization is emerging as a powerful computational tool for the targeted formulation of multicomponent supramolecular systems. By applying Bayesian optimization to map assembly landscapes, optimize covalent modification, and tailor supramolecular networks, we have demonstrated that Bayesian optimization can be easily integrated into established experimental workflows in the field. Its versatility and effectiveness in guiding the design and optimization of multicomponent supramolecular systems, while reducing the reliance on prior knowledge or expert intuition, highlights the advantage of data-driven approaches in addressing multidimensional challenges. While the case studies presented here focus on two-dimensional optimization tasks, the underlying methodology is readily extendable to higher-dimensional design spaces, particularly when integrated with automated experimentation that enables larger and more diverse data sets. In general, Bayesian optimization requires quantitative characterization techniques that are both fast and reliable to achieve time-efficient results. Furthermore, it is most effective when the property of interest can be directly characterized. It should be noted that Bayesian optimization can converge more slowly or become trapped in local optima if the initial sampling is poorly distributed or when the experimental noise is high. Monitoring convergence indicators should be considered to help detect when further iterations become inefficient. With these considerations in mind, we anticipate that Bayesian optimization and other methods of machine learning-guided experimentation will become the standard approach for design space exploration of complex supramolecular systems.

## Supplementary Material



## Data Availability

Python scripts that allow for reproduction of simulated Bayesian optimization runs and scripts that were used for experimental Bayesian optimization runs are available via Zenodo at: 10.5281/zenodo.14235422.

## Notes

Additional UV–vis and CD spectra, additional rheology measurements, details of the thermodynamic model, additional analyses of Bayesian optimization performance and additional phase diagrams (PDF)
