# Supplementary material for: Bayesian Optimization for Multicomponent Supramolecular Systems
Source: J Am Chem Soc. 2025 Sep 4;147(37):33607–14. doi: 10.1021/jacs.5c08539 (PMC12447505; doi:10.1021/jacs.5c08539)
Supplement: Supplementary file 1 [file ja5c08539_si_001.pdf]

Supplementary information for:

Bayesian optimization for multicomponent supramolecular systems

Stef A.H. Jansen,[1,2] Albert J. Markvoort,[1,3] Freek V. de Graaf,[1,2] Martin G.T.A. Rutten,[1] Patricia Y.W. Dankers,[1,4] Ghislaine Vantomme,[1,2] Tom F.A. de Greef[1,3,4,5,6]* and E.W. Meijer[1,2,7,8]*

[1]Institute for Complex Molecular Systems, [2]Laboratory of Macromolecular and Organic Chemistry, [3]Synthetic Biology Group, [4]Laboratory of Chemical Biology, Eindhoven University of Technology, 5600 MB Eindhoven, The Netherlands.
[5]Institute for Molecules and Materials, Radboud University, 6500 GL Nijmegen, The Netherlands.
[6]Center for Living Technologies, Eindhoven-Wageningen-Utrecht Alliance, 3584 CS Utrecht, The Netherlands.
[7]School of Chemistry and RNA Institute, University of New South Wales, 2052 Sydney, Australia.
[8]Max Planck Institute for Polymer Research, Ackermannweg 10, 55128 Mainz, Germany.

*To whom correspondence should be addressed: t.f.a.d.greef@tue.nl, e.w.meijer@tue.nl

Contents

S1. Methods S3
S1.1 Materials S3
S1.2 Sample preparation S3
S1.3 Measurement methods S4
S1.4 Bayesian optimization methods S5
S2. Molecular structures and syntheses of monomers S9
S2.1 **(*S*)-Por$_{Zn}$** S9
S2.2 **a-BTA** S9
S2.3 **(*R*)-Glu-BTA** S10
S2.4 **(*S*)-** and **(*R*)-Glu(OMe)-BTA** S10
S2.5 **UPy-EG$_{11}$** S10
S2.6 **Phz-EG$_4$** S11
S2.7 **BTA-EG$_4$** S11
S3. Supplementary figures S12
Table S1 and **Figure S8:** Statistical analysis of assembly landscape optimization S12
Figure S9: Medians of required experiments for different batch sizes and data errors S12
Table S2 and **Figure S10:** Statistical analysis of covalent modification optimization S14
Figure S11: Dependency on initial parameters for covalent modification optimization S15
Figure S12: Performance of individual optimization runs for covalent modification S15
Figure S13: CD experimental data of mixtures before and after covalent modification S16
Figure S14: Assembly landscape of **UPy-EG$_{11}$** with **OTAB** S16
Figure S15: Assembly landscape of **BTA-EG$_4$** with **OTAB** S17
Figure S16: Assembly landscapes to study non-orthogonality of **Phz-EG$_4$** S17
Figure S17: Rheological measurements of dilution series S18
Figure S18–S20: Additional data of rheological measurements S18
S4. Details on computations with mass-balance models S20
S4.1 Assembly landscape simulations S20
S4.2 Diluted Majority-rules simulations for covalent copolymer modification S22
S5. Python scripts used for Bayesian optimization S25
S6. References S26

S1. Methods

S1.1 Materials

All chemicals used were obtained from commercial suppliers and used without prior purification. Spectroscopic grade methylcyclohexane (MCH) was used. Water was purified on an EMD Millipore Milli-Q Integral Water Purification system.

S1.2 Sample preparation

Sample preparation for UV/Vis spectroscopy samples assembly landscapes: Spectroscopic samples were prepared by weighing the desired compound into a screw-capped vial and adding the required amount of MCH to reach the desired concentration. The sample was then sonicated for approximately 1 minute and vortexed for 20 seconds to fully dissolve the compounds. In a separate vial, the desired alcohol was mixed with MCH, and the solution was vortexed for 20 seconds. The samples were then prepared for measurement by pipetting the monomer solution, alcohol solution and MCH into quartz cuvettes with a pathlength of 1 or 10 millimeter. A pathlength of 1 millimeter was used for concentrations above 10 μM (and the signal was then multiplied by 10), and a pathlength of 1 centimeter for concentrations below 10 μM. Gilson Microman pipettes were used to transfer solutions. The samples were then vigorously shaken and inserted in the instrument. Then, the samples were thermally equilibrated in the instrument at 70 °C and subsequently cooled with a rate of 0.5 °C/min to the temperature selected by the BO algorithm.

Sample preparation for CD spectroscopy samples BTA copolymers: Stock solutions of **(*R*)-Glu-BTA**, **(*R*)-Glu(OMe)-BTA**, **(*S*)-Glu(OMe)-BTA** and **a-BTA** were prepared by weighing the solid materials, followed by addition of MCH to get a stock solution of 200 μM for **(*R*)-Glu-BTA** and, **(*R*)-** and **(*S*)-Glu(OMe)-BTA** and 1 mM for **a-BTA**. The stock solutions were placed in a sonication bath for 10 minutes, subsequently heated to 80 °C and cooled down to room temperature to get homogeneous solutions. In a 1 cm pathlength cuvette, the BTA stock solutions were mixed with MCH to obtain a final concentration of 50 μM after addition of methanol. For the newly measured ΔCD optimum, the sample composition was 0.752 / 0.119 / 0.129 (**a-BTA** / **(*R*)-Glu-BTA** / **(*S*)-Glu(OMe)-BTA**) in the first mixture, with the **(*R*)-Glu-BTA** substituted for **(*R*)-Glu(OMe)-BTA** in the second mixture. Gilson Microman pipettes were used to transfer solutions. Experiments were conducted at room temperature.

Sample preparation for UV/Vis spectroscopy samples monomers with surfactants: For each monomer, a stock solution was made with the maximum surfactant and monomer concentrations as selected by the user. For **UPy-EG$_{11}$**, this was 1 M **OTAB** with 100 mM **UPy-EG$_{11}$**. For **Phz-EG$_4$**, this solution contained 1 M **OTAB** and 75 mM **Phz-EG$_4$**. For **BTA-EG$_4$**, the concentrations were 400 mM **OTAB** and 120 mM **BTA-EG$_4$**. The **UPy-EG$_{11}$** stock solution was made by adding 0.1 M NaOH in MQ-H_2O to the weighed **OTAB**, heating until dissolved, and then adding the required amount of this solution to the weighed **UPy-EG$_{11}$**. The solution was then heated and stirred at 80 °C in a water bath for 30 minutes, after which 1/9 vol% 1 M HCl in MQ-H_2O was added. The mixture was vigorously shaken and then left in the unplugged water bath until it reached room temperature. The stock solutions of **BTA-EG$_4$** and **Phz-EG$_4$** were made by adding MQ-H_2O to the weighed **OTAB**, heating it until dissolved, and then adding the required amount of this solution to the weighed monomer, with subsequent heating until dissolved. To prepare samples selected by the BO algorithm, first a solution of **OTAB** at the maximum concentration in MQ-H_2O was added and then MQ-H_2O was added to obtain the desired conditions. The mixture was vigorously shaken and left for 2 minutes after which it was pipetted into a cuvette with a pathlength of 0.1 (**UPy-EG$_{11}$** and **Phz-EG$_4$**) or 0.01 (**BTA-EG$_4$**) mm. Gilson Microman pipettes were used to transfer the samples.

Sample preparation for rheology: Samples for rheology were prepared by weighing the required amount of each compound into separate vials. 0.1 M NaOH in MQ-H_2O was added to the weighed **OTAB**. The required amount of this solution was then added to the weighed **BTA-EG$_4$** to obtain the desired concentration. This solution was added to the weighed **Phz-EG$_4$**, which was subsequently added to the **UPy-EG$_{11}$**. Before each step, the solution was heated and vortexed until well-dissolved. Then,

the **UPy-EG$_{11}$** was heated and vortexed, and afterwards stirred at 80 °C in a water bath for 30 minutes. 1/9 vol% of 1 M HCl in MQ-H_2O was added, the mixture was vigorously shaken and then left in the unplugged water bath until it reached room temperature and aged overnight. This stock solution was then diluted with MQ-H_2O to the desired concentration, and vortexed until homogeneous. The samples were pipetted with Gilson Microman pipettes into a 96-well plate, which was covered and sealed with Parafilm. Afterwards, the samples were measured on the same day.

S1.3 Measurement methods

UV/Vis spectroscopy: UV/Vis spectroscopy was performed on a JASCO V-650 spectrophotometer with a JASCO ETCR-762 temperature controller.

Converting UV/Vis absorption to degree of aggregation: For the **(*S*)-Por$_{Zn}$** assembly landscapes, the samples were thermally equilibrated in the instrument at 70 °C and subsequently cooled with a rate of 0.5 °C/min to the temperature selected by the BO algorithm. The UV/Vis absorption at λ=393 nm was monitored, and the absorption was divided by the concentration to obtain the molar extinction coefficient of the sample $\varepsilon_{\text{sample}}$. The molar extinction coefficient of the sample at 70 °C was subtracted to get the $\Delta\varepsilon$. Finally, this was converted to the degree of aggregation by dividing it by the $\Delta\varepsilon$ of a sample in the fully polymerized state ε_P compared to the fully depolymerized state ε_M:

$$\text{degree of aggregation} = \frac{\varepsilon_{\text{sample}} - \varepsilon_{\text{sample,70}}}{\varepsilon_{\text{P}} - \varepsilon_{\text{M}}} \qquad \text{(S1)}$$

Converting UV/Vis absorption to degree of network formation: For the network transitions of water-soluble supramolecular polymers, the samples were inserted in the measurement instrument and the UV/Vis at a diagnostic wavelength was collected. The selected diagnostic wavelengths were λ=270 and 230 nm for **UPy-EG$_{11}$** and **BTA-EG$_4$**, respectively. For **Phz-EG$_4$**, the difference between the UV/Vis absorption at λ=250 and 255 nm was used, as there appeared to be no single diagnostic wavelength that gave reliable results. Similarly, for **Phz-EG$_4$ + BTA-EG$_4$**, the difference between the UV/Vis absorption at λ=205 and 230 nm was used. The UV/Vis signal at this wavelength was divided by the concentration of the sample c to obtain the molar extinction coefficient of the sample $\varepsilon_{\text{sample}}$. The change in molar extinction coefficient was calculated as $\Delta\varepsilon = \varepsilon_{\text{sample}} - \varepsilon_M$, which was then multiplied by the concentration to get the change in absorption caused by polymerization:

$$\Delta\text{UV}_{\text{pol}} = c \cdot \Delta\varepsilon = c \cdot (\varepsilon_{\text{sample}} - \varepsilon_{\text{M}}) \qquad \text{(S2)}$$

The $\Delta\text{UV}_{\text{pol}}$ of each sample was the compared to the $\Delta\text{UV}_{\text{pol}}$ of a sample at the gelation concentration of the respective monomer. The gelation concentrations were approximately 27, 8 and 8 mM for **UPy-EG$_{11}$**, **Phz-EG$_4$**, and **BTA-EG$_4$**, respectively. The $\Delta\text{UV}_{\text{pol}}$ of the samples were divided by $2\cdot\Delta\text{UV}_{\text{pol}}$ of the gelation reference to obtain the degree of network formation, so that a value above 0.5 represented a network that could support a self-standing gel, and a value below 0.5 indicated that the network could support a self-standing gel.

This approach assumes approximate linearity between UV/Vis absorption and network density but is certainly not an exact determination of the gelation boundary. For **Phz-EG$_4$** specifically, the use of a differential method ($\Delta A_{250\text{–}255}$) compensates for the lack of a clear diagnostic peak but may introduce additional uncertainty near the transition region. Ideally, the degree of network formation would be characterized with direct rheological measurements during optimization, but this was impractical in the scope of this study due to time- and equipment constraints. Therefore, this UV/Vis spectroscopy-based method provides a practical and consistent trend for comparing relative network formation under different conditions.

Circular Dichroism (CD) spectroscopy: CD spectroscopy was performed on a JASCO J-815 CD spectropolarimeter with a JASCO MPTC-490S thermostat.

Converting CD signals to ΔCD: For the BTA copolymers, the samples were thermally equilibrated at 85 °C in the instrument and subsequently cooled with a rate of 1 °C/min to 20 °C. Then the CD spectra were collected and the ΔCD at λ=222 nm was calculated by the difference between the component mixture that would be present after 100% conversion of **(*R*)-Glu-BTA (a-BTA+(*R*)-Glu(OMe)-**

BTA+(*S*)-Glu(OMe)-BTA) and the mixture before any conversion (**a-BTA+(*R*)-Glu-BTA+(*S*)-Glu(OMe)-BTA**).

Rheology: Rheological measurements were carried out on a TA Instruments Dynamic Hybrid Rheometer 3 (DHR-3) in a 20 mm aluminum cone-plate (2.007°) geometry with a truncation gap of 56 μm. A solvent trap was used to minimize sample drying. Samples were loaded at 20 °C and allowed to equilibrate for 100 s while performing an oscillatory time sweep with amplitude $\gamma = 0.01\%$ at frequency $\omega = 1$ rad·s^{-1}. Frequency sweep measurements were performed at $\omega = 100$ rad·s^{-1} to 0.1 rad·s^{-1}, at a strain of $\gamma = 0.01\%$. Alternating strain ($\gamma = 0.01\%$ or $\gamma = 10\%$) experiments were performed with a fixed frequency of $\omega = 1$ rad·s^{-1}. The initial strain ($\gamma = 0.01\%$) was applied for 30 s while monitoring the complex modulus G*, after which the strain was changed ($\gamma = 10\%$). This alternating change in strain amplitude was applied for 6 circles to investigate self-healing behavior.

S1.4 Bayesian optimization methods

Defining the design space

The Bayesian optimization framework requires the specification of the design space boundaries by the user. In the case of the assembly landscape of **(*S*)-Por$_{Zn}$** with 2480 equivalents EtOH in MCH, the temperature ranged from 10 to 70 °C and the **(*S*)-Por$_{Zn}$** concentration from 1 to 50 μM. For the covalent modification of BTA copolymers, the sergeant excess ranged from -1.0 to 1.0 and the sergeant fraction from 0 to 100 %. For the **UPy-EG$_{11}$/OTAB** mixtures, the **UPy-EG$_{11}$** equivalents ranged from 0.02 to 0.10 and the **OTAB** concentration from 300 to 1200 mM. For the **Phz-EG$_4$/OTAB** mixtures, the **Phz-EG$_4$** equivalents ranged from 0.005 to 0.066 and the **OTAB** concentration from 100 to 1000 mM. For the **BTA-EG$_4$/OTAB** mixtures, the **BTA-EG$_4$** equivalents ranged from 0.05 to 0.30 and the **OTAB** concentration from 25 to 300 mM. These sampling ranges were selected based on previous experimental observations and solubility constraints, ensuring the parameter space likely contained the phase transition or assembly boundary.

Surrogate model for prediction

For the prediction step in the Bayesian optimization cycle, Gaussian process regression (GPR) was used as the surrogate model, which was performed with the scikit-learn module in Python. The sum of a constant kernel with a Matern 5/2 kernel and a white noise kernel was used.[S1] This kernel was chosen to balance flexibility for modeling moderately smooth assembly landscapes with robustness to experimental noise. In preliminary tests, we compared this to alternative kernels (e.g., RBF, Matern 3/2) and observed no consistent improvement in prediction accuracy for these data. Each length scale parameter of the Matern kernel was constrained between the minimum and the maximum value in the design space for the corresponding variable. For parameters that cannot be negative (e.g. concentrations), the prediction from the GPR was set to zero if a negative value was predicted for a set of variables. For parameters that cannot be larger than unity (e.g. degree of aggregation), the prediction from the GPR was set to unity if a value larger than unity was predicted for a set of variables. The mean and standard deviation of the GPR posterior predictive were then inserted into the acquisition function to select the next data points.

Acquisition functions and minimization

From the GPR posterior predictive, μ is the mean and σ the standard deviation at data point x. The following acquisition functions α were minimized to select the next experiments:

- Random:
  Random sampling was performed in Python with random.default_rng from the NumPy module.

- Exploitative[S1]:

$$\alpha(x) = |\mu(x) - \text{ target}| \tag{S3}$$

  Minimizing the exploitative acquisition function will target the values of interest, selecting the data point x of which $\mu(x)$ from the GPR posterior predictive is closest to the target value.

- Explorative[S1]:

$$\alpha(x) = -\sqrt{\sigma(x)} \tag{S4}$$

  Minimizing the explorative acquisition function will target the data point x with the highest $\sigma(x)$ of the GPR posterior predictive.

- Hybrid[S1]:

$$\alpha(x) = \frac{-\sqrt{\sigma(x)}}{|\mu(x) - \text{ target}| + \varepsilon} \tag{S5}$$

with ε being a parameter for tuning the balance of explorative and exploitative sample selection. Typical values for ε are between 0 and 1. For the hybrid acquisition function, ε was set to 0.5 (unless mentioned otherwise) to balance exploration and exploitation based on test runs for this system. We note that this parameter is a good starting point for applications to different systems but should likely be adjusted to maximize performance. Minimization of the acquisition function will target data points x of which the $\mu(x)$ from the GPR posterior predictive is close to the target value and the $\sigma(x)$ is high.

- Expected improvement (EI)[S2]:

$$\alpha(x) = \begin{cases} (\mu(x) - f(x^+) - \varepsilon)\Phi(Z) + \sigma(x)\phi(Z) & \text{if } \sigma(x) > 0 \\ 0 & \text{if } \sigma(x) = 0 \end{cases} \tag{S6}$$

with

$$Z = \begin{cases} \dfrac{\mu(x) - f(x^+) - \varepsilon}{\sigma(x)} & \text{if } \sigma(x) > 0 \\ 0 & \text{if } \sigma(x) = 0 \end{cases} \tag{S7}$$

where $f(x^+)$ is the optimum from the currently sampled data, which is located at position x^+. Φ and ϕ are the cumulative distribution function and probability density function of the standard normal distribution, respectively. ε determines the balance of explorative and exploitative sample selection, with higher values favoring exploration. We used a value for ε of 0.01. EI is an effective acquisition function for optimizations, hence we used it to find the optimal composition for covalent modification of BTA copolymers.

- ε-Greedy:

$$\alpha(x) = -|\mu(x)| \tag{S8}$$

with a chance of ε to select a random point. Minimization the ε-Greedy acquisition function will target data point x with the highest values for $\mu(x)$ from the GPR posterior predictive, while selecting a random sample occasionally for explorative purposes.

- Thompson sampling (TS):

$$\alpha(x) = -|g(x)| \tag{S9}$$

where $g(x)$ is a drawn sample from the GPR posterior predictive. Drawing the samples contributes to the explorative aspect of this acquisition function.

The acquisition functions were minimized within the user-specified variable boundaries using the minimized function from the SciPy module. The minimization of the function was started from 100 random initial data points and the lowest obtained value from these 100 minimizations was selected as the best sample.

Simulated experiments with mass-balance models

To analyze the BO framework, simulated experiments were used for the assembly landscapes of porphyrins and the modification for BTA copolymers. The mass-balance models used for these simulations are derived in Section S4 of the supplementary information.

Selecting multiple samples in one batch

When multiple samples were selected per iteration, the first sample of a batch was selected according to the minimization described above. A gaussian function was then added to the acquisition function with the maximum on the position of the previously selected data point to penalize the selection of comparable samples. The width of the gaussian was chosen as a fraction of the width of the design space in each dimension. This function was then minimized to select the next data point, after which another gaussian function was added with its maximum on the position of the newly sampled data point. This was repeated until the user-defined batch size was obtained.

Termination of optimization

The total number of iterations for each case study was determined by practical convergence (when further sampling no longer significantly changed the prediction) or set to a fixed value (e.g., 50 for the helicity simulation) to allow consistent comparison between acquisition functions.

Performance assessment

The performance of Bayesian optimization of assembly landscapes required the comparison of two surfaces. To quantify the similarity of the two surfaces, we calculated the R^2 of the GPR prediction with respect to the simulated ground truth:

$$R^2 = 1 - \frac{\sum_x (f_x - \mu(x))^2}{\sum_x (f_x - \overline{f})^2} \qquad \text{(S10)}$$

where f contains a 200x200 grid of data points x from the ground truth and μ is the mean of the GPR prediction.

Statistics methods

To assess the performance and robustness of the BO for the simulated optimizations of assembly landscapes of **(*S*)-Por$_{\mathbf{Zn}}$** monomers, we performed 100 independent optimizations (with 50 iterations each) per acquisition function and compared the resulting R^2 distributions. Denoting the resulting R^2-values for a certain acquisition function as y_i with i between 1 and n (the number of optimizations, here 100), we calculate the average loss (μ_{loss}), the standard deviation (σ) and the worst-case loss (ξ) as follows.

The average loss is defined as the mean difference between the global maximum y^+ and the resulting R^2-values of the n simulations (y_i), i.e.

$$\mu_{\text{loss}} = \frac{1}{n}\sum_{i=1}^{n}(y^+ - y_i) \qquad \text{(S11)}$$

In this case of R^2-values, for the global maximum y^+ we simply take $y^+ = 1$, i.e., perfect reconstruction of the ground truth.

The standard deviation

$$\sigma = \sqrt{\frac{1}{n}\sum_{i=1}^{n}(y_i - \bar{y})^2} \qquad \text{(S12)}$$

with $\bar{y}$ the mean of the resulting R^2-values of the n simulations (y_i), is a measure of the variation in optimization outcome over n optimizations.

The worst-case loss

$$\xi = \max_{1 \le i \le n} (y^+ - y_i) \tag{S13}$$

is the maximum difference between the global maximum (y^+) and the resulting R^2-values of the n simulations (y_i).

The results for the various acquisition functions are summarized in Table S1. Furthermore, we performed one-sample t-tests on the R^2-values of the n independent optimizations after x iterations to test whether the mean of the R^2-values after x iterations reached a specific threshold. The results for threshold 0.9 is shown in Figure S8.

To assess the performance and robustness of the BO for the simulated optimizations of covalent modification of supramolecular polymers, we performed 50 independent optimizations (with 50 iterations each) per acquisition function and compared the average loss, the standard deviation and the worst-case loss according to above formulae, where now y_i is the resulting ΔCD of the i-th optimization and y^+ the global maximum ΔCD obtained. The results are summarized in Table S2. Furthermore, Welch's t-tests were performed to compare distinguishability of the mean ΔCD after x iterations for the Expected Improvement acquisition function with the mean ΔCD after the same number of iterations for another acquisition function. The results for after 50 iterations are shown in Table S2 and Figure S9 shows the results as a function of the iteration number.

S2. Molecular structures and syntheses of monomers

S2.1 **(*S*)-Por$_{Zn}$**

The synthesis of **(*S*)-Por$_{Zn}$** was reported in reference S3.

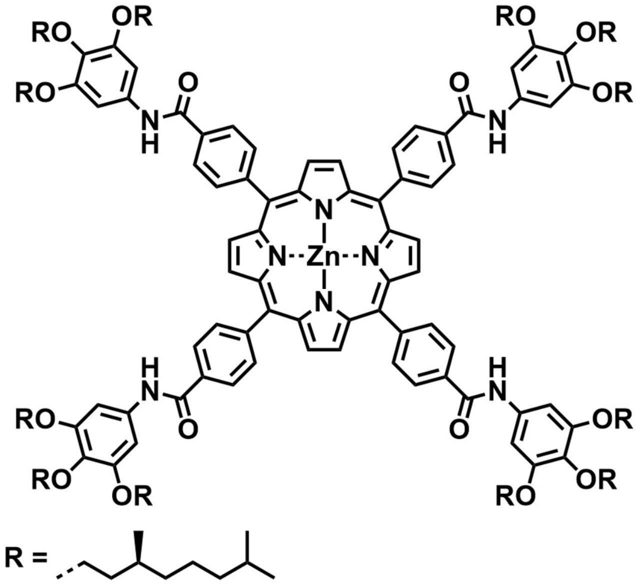

Figure S1: Molecular structure of **(*S*)-Por$_{Zn}$**.

S2.2 **a-BTA**

The synthesis of **a-BTA** was reported in reference S4.

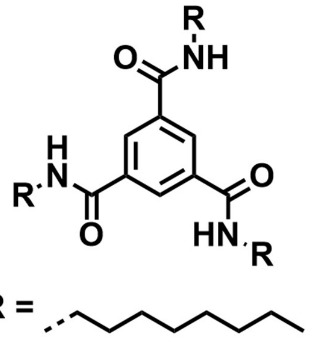

Figure S2: Molecular structure of **a-BTA**.

S2.3 **(*R*)-Glu-BTA**

The synthesis of **(*R*)-Glu-BTA** was reported in reference S5.

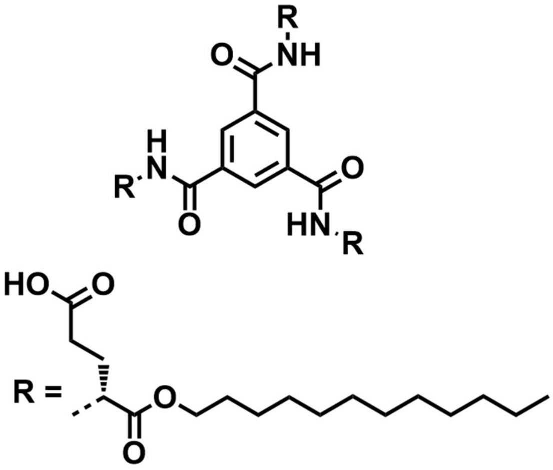

Figure S3: Molecular structure of **(*R*)-Glu-BTA**.

S2.4 **(*S*)-** and **(*R*)-Glu(OMe)-BTA**

The synthesis of **(*S*)-** and **(*R*)-Glu(OMe)-BTA** was reported in reference S5.

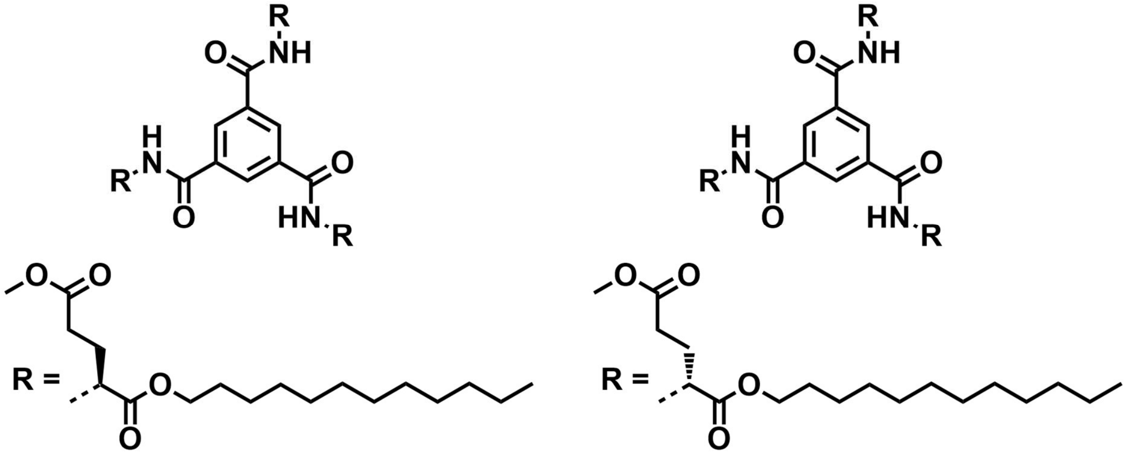

Figure S4: Molecular structure of **(*S*)-** and **(*R*)-Glu(OMe)-BTA** (left and right, respectively).

S2.5 **UPy-EG$_{11}$**

The synthesis of **UPy-EG$_{11}$** was reported in reference S6.

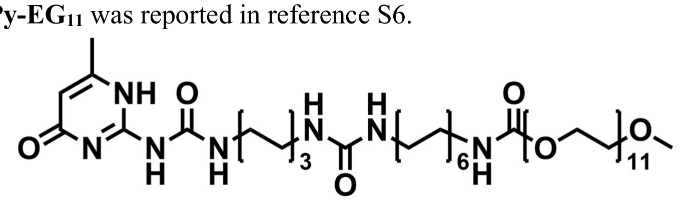

Figure S5: Molecular structure of **UPy-EG$_{11}$**.

S2.6 **Phz-EG$_4$**

The synthesis of **Phz-EG$_4$** was reported in reference S7.

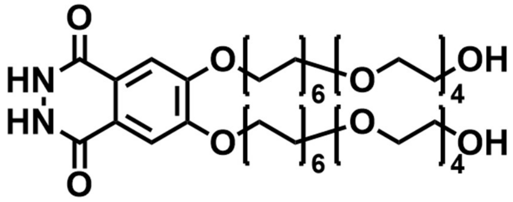

Figure S6: Molecular structure of **Phz-EG$_4$**.

S2.7 **BTA-EG$_4$**

The synthesis of **BTA-EG$_4$** was reported in reference S8.

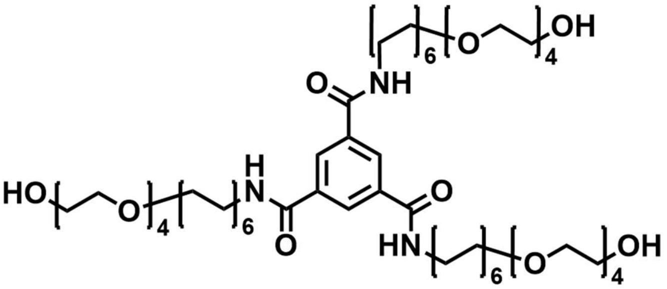

Figure S7: Molecular structure of **BTA-EG$_4$**.

S3. Supplementary figures

Table S1: Statistical analysis of 100 simulated optimizations of assembly landscapes of **(*S*)-Por$_{Zn}$** monomers with different acquisition functions after 50 iterations. For each acquisition function, the average loss (μ_{loss}), standard deviation (σ), and the worst-case loss (ξ) in maximum achieved R^2. The results show that the hybrid acquisition function with $\varepsilon = 0.5$ is the most robust, having the lowest worst-case loss and average loss, and having a low standard deviation.

	Exploitative	Explorative	Hybrid $\varepsilon = 0.1$	Hybrid $\varepsilon = 0.3$	Hybrid $\varepsilon = 0.5$	Hybrid $\varepsilon = 0.7$	Random
μ_{loss}	0.029	0.022	0.018	0.014	0.013	0.014	0.028
σ	0.040	0.007	0.013	0.012	0.008	0.012	0.012
ξ	0.086	0.039	0.054	0.038	0.038	0.047	0.059

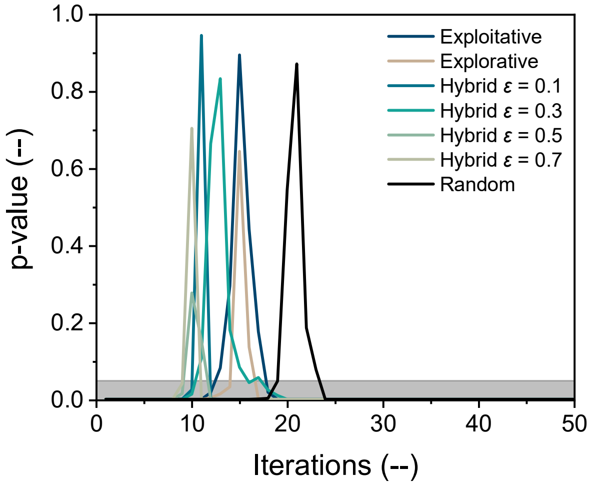

Figure S8: One-sample *t*-test p-values for the hypothesis $R^2 = 0.9$ monitored at each iteration during the optimizations of assembly landscapes. In the first 7 iterations, the p-values are below the threshold of 0.05 for statistical distinguishability (marked in grey), because all optimizations started with an R^2 well below 0.9. With an increasing number of iterations, a spike is visible for each acquisition function that indicates when the mean R^2 can no longer be statistically distinguished from 0.9 anymore. The hybrid acquisition function with $\varepsilon = 0.5$ or 0.7 achieves this in the least number of iterations. Upon further optimization, the predicted landscapes converge to the ground truth and the p-values go down again because the mean R^2 then well exceeds 0.9.

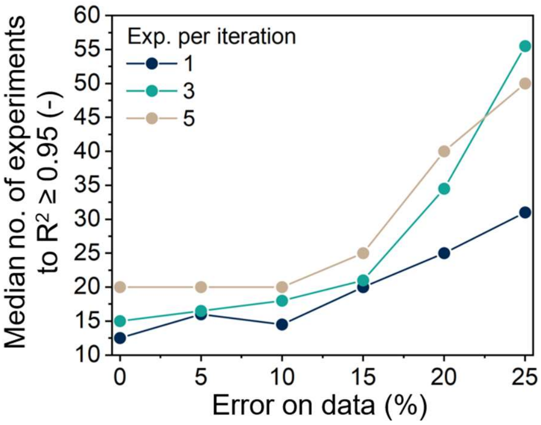

Figure S9: Median number of experiments required to reach the target R^2 of 0.95 for different batch sizes (1, 3, 5) and noise levels (up to 25% error). Compared to the mean values shown in Figure 2e, the medians indicate that the higher average for a batch size of 3 at 25% error was caused by a few outlier runs. This suggests that the apparent difference is not statistically robust and that typical performance is similar across batch sizes at high error.

Table S2: Statistical analysis of 50 simulated optimizations of the covalent modification of supramolecular polymers with different acquisition functions after 50 iterations. For each acquisition function, average loss (μ_{loss}), standard deviation (σ), and worst-case loss (ξ) in maximum achieved ΔCD are given as well as the p-values of Welch's *t*-tests for the null hypothesis of equal average performance to expected improvement (p). These results show that the acquisition functions are statistically indistinguishable (except for the explorative acquisition function), but that Expected Improvement (EI) is the most robust, having the lowest worst-case loss, average loss and standard deviation.

	Expected Improvement (EI)	ε-Greedy $\varepsilon = 0.1$	Exploitative	Explorative	Thompson sampling (TS)
μ_{loss}	0.004	0.052	0.848	0.806	0.026
σ	0.009	0.267	4.921	0.578	0.182
ξ	0.063	1.755	34.625	2.012	1.287
p	1.000	0.205	0.231	<0.001	0.387

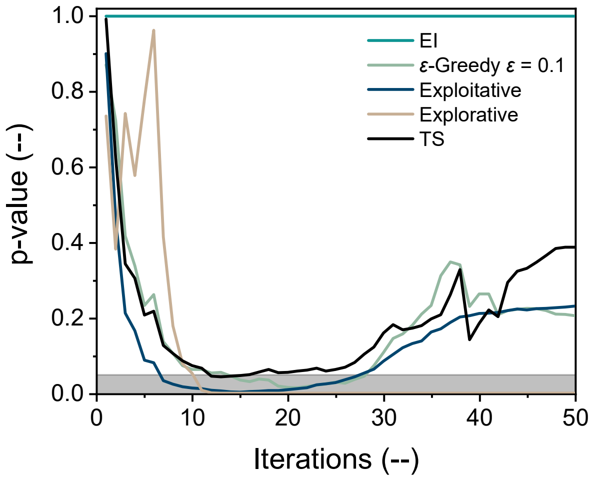

Figure S10: Welch's *t*-test p-values for the hypothesis of equal average performance (maximum achieved ΔCD) to Expected Improvement, monitored during the optimizations of covalent modification of supramolecular polymers. In the first iterations, the randomness of the initial parameter sets results in high p-values for all acquisition functions, but EI outperforms all acquisition functions at some point during the optimization (p-values < 0.05, in the area marked in grey). As optimizations continue and converge to the optimum, the p-values gradually increase again and the achieved ΔCD becomes again statistically indistinguishable from that obtained with EI in the same number of iterations.

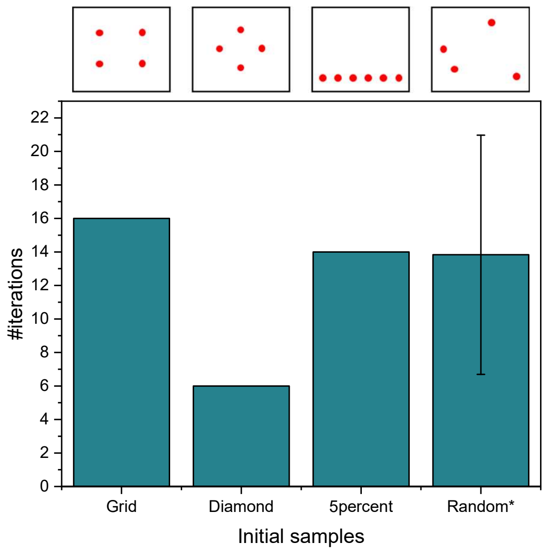

Figure S11: Number of iterations to reach the maximum simulated ΔCD, starting with 4 data points in a grid or diamond, starting from the 5 mol% sergeant line (which was investigated experimentally in reference S3), or 4 random data points (average of 6 different random sets of data points). The optimizations were performed with simulated data. These results show that the optimization is completed in 13 to 16 iterations on average for all methods, except for the 4 initial data points in a diamond. The initial sampling in a diamond was beneficial for this particular phase diagram due to the position of the optimum compared to the initial points and is not expected to outperform the other initial sampling methods for different phase diagrams.

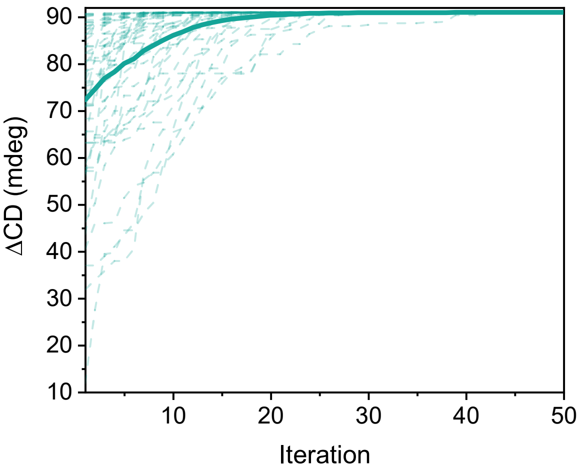

Figure S12: Performance plot (monitoring the simulated change in CD signal upon covalent modification of the sergeant) of 50 optimization runs (dashed lines) and the average (solid line). The optimizations were performed with the expected improvement acquisition function and with simulated data. The optimizations show a widespread in the early stages below 10 iterations, due to the different random initial data points that are selected in each run. Nonetheless, most iterations converge to the maximum ΔCD in 25 iterations, underlining the robustness of the optimization with the expected improvement function.

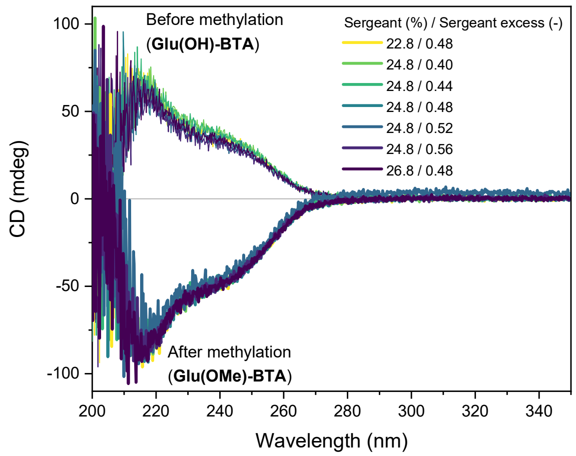

Figure S13: CD data of mixtures with composition that would be obtained before (**a-BTA+(*R*)-Glu-BTA+(*S*)-Glu(OMe)-BTA**) and after covalent modification with 100% conversion (**a-BTA+(*R*)-Glu(OMe)-BTA+(*S*)-Glu(OMe)-BTA**). Different samples around the computationally found optimum (24.8 sergeant mol% and 0.48 (*R*)-sergeant excess) were measured. The change in CD signal of the optimum was plotted in Figure 3f. These data show that the phase diagram is smooth around the newly found optimum, as most of the proximal samples give similar CD signals. Therefore, it is difficult to experimentally confirm that 24.8 sergeant mol% and 0.48 (*R*)-sergeant excess is the true global optimum.

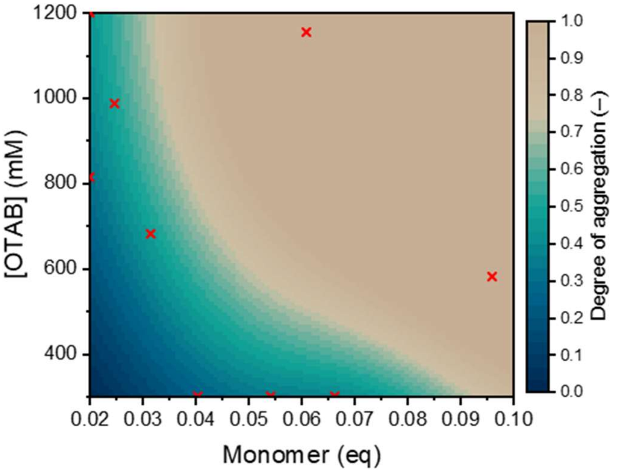

Figure S14: Posterior mean or predicted phase diagram of **UPy-EG$_{11}$** with **OTAB** in water, after 10 experiments selected with BO. Red crosses indicate the experimentally measured data points. **OTAB** did not induce a significant change in **UPy-EG$_{11}$** assembly, as the mixtures did not show a solution state at high concentrations.

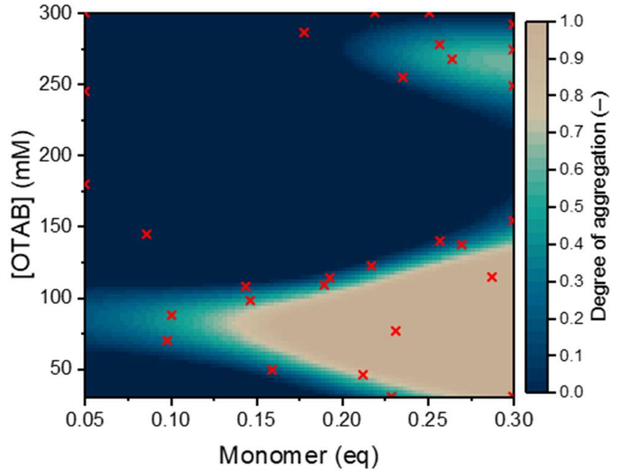

Figure S15: Posterior mean or predicted phase diagram of **BTA-EG$_4$** with **OTAB** in water, after 30 experiments selected with BO. Red crosses indicate the experimentally measured data points. The area with higher degree of aggregation around 275 mM **OTAB** and 0.30 monomer equivalents was an artifact of the data normalization method, where the interfering absorbance of the **OTAB**. In these samples, no gels or high viscosities were observed macroscopically. The mixtures show a solution-hydrogel-solution transition upon dilution above 0.13 equivalents of **BTA-EG$_4$** with respect to **OTAB**.

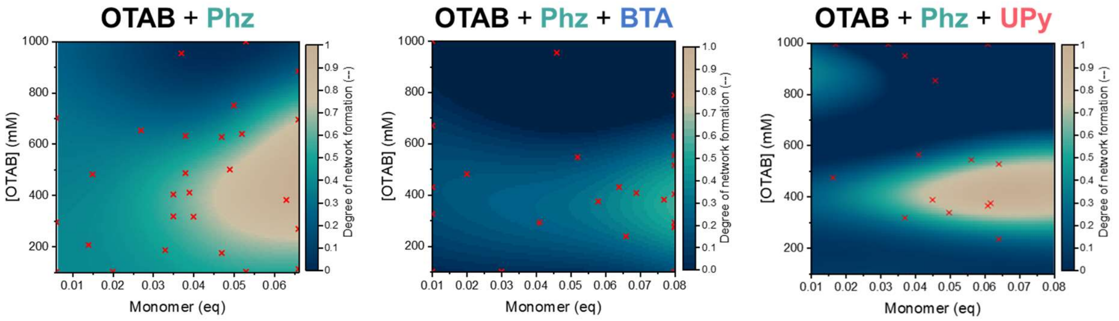

Figure S16: Phase diagrams of **Phz-EG$_4$** with **OTAB** (left), **OTAB** and 0.19 eq **BTA-EG$_4$** (middle), and **OTAB** and 0.03 eq **UPy-EG$_{11}$** (right). The x-axis shows the equivalents of **Phz-EG$_4$** compared to **OTAB**. Note that the optimizations with **BTA-EG$_4$** and **UPy-EG$_{11}$** were only performed for a few iterations to get the general position of the gel phase, and that more iterations are required to obtain accurate phase diagrams. Nonetheless, these results show that the gel phase of **Phz-EG$_4$** is significantly shifted to the right in the presence of **BTA-EG$_4$** (> 0.07 eq **Phz-EG$_4$** required), while a gel is formed in a similar **Phz-EG$_4$** eq range (>0.04 eq) in the presence of **UPy-EG$_{11}$**. These results indicate that **BTA-EG$_4$** and **Phz-EG$_4$** interact, confirming the non-orthogonality of the supramolecular networks.

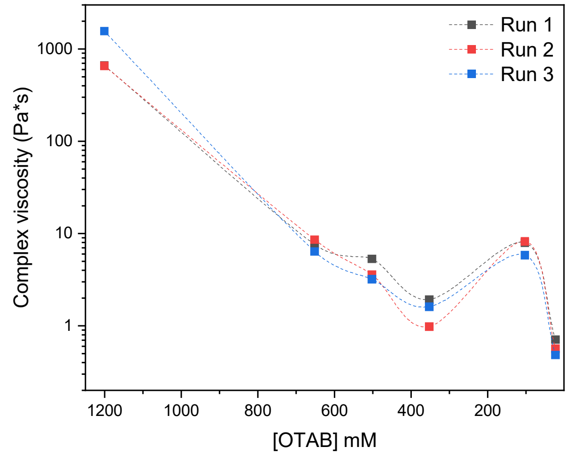

Figure S17: Rheological measurements of three dilution series of the **OTAB**:**UPy-EG$_{11}$**:**Phz-EG$_4$**:**BTA-EG$_4$** system (1:0.024:0.055:0.160), of which the average is shown in Figure 4e. Measurements were conducted at 20 °C at ω = 1 rad·s^{-1} and γ = 1%.

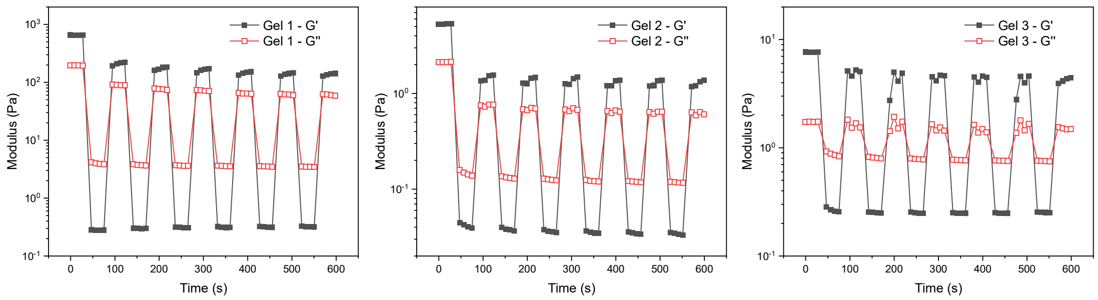

Figure S18: Step-strain rheological measurements in which the oscillatory strain (γ) alternated between 1% and 1000% for 30-s periods (ω = 1 rad·s^{-1}). Results are shown of the three gels/viscous phases of the **OTAB**:**UPy-EG$_{11}$**:**Phz-EG$_4$**:**BTA-EG$_4$** system (1:0.024:0.055:0.160) in dilution series Run 1 of Figure S16. Gel 1, 2 and 3 are at **OTAB** concentrations of 1200, 500 and 100 mM, respectively. At low strains an elastic-like gel is formed (G' > G'') while high strains result in liquification of the material (G'' > G'). Alternating between high and low strains allows the rapid switch between a solution and gel. All three phases show fast and almost complete self-healing properties.

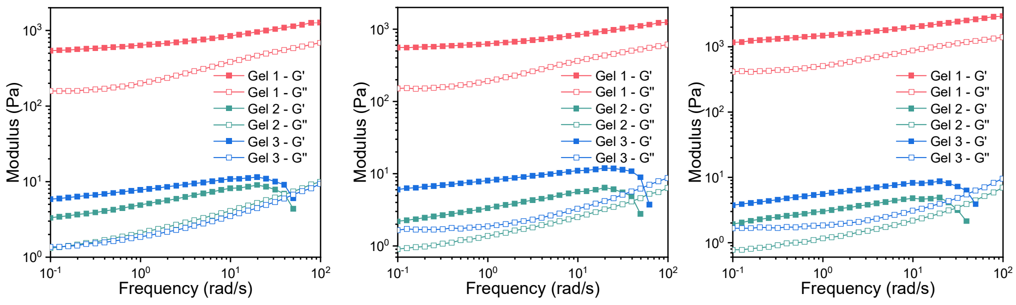

Figure S19: Additional frequency-dependent rheological measurements of the three gels/viscous phases of the **OTAB**:**UPy-EG$_{11}$**:**Phz-EG$_4$**:**BTA-EG$_4$** system (1:0.024:0.055:0.160) in dilution series of Figure S16 (γ=1%, from left to right Run 1, 2 and 3). Gel 1, 2 and 3 are at **OTAB** concentrations of 1200, 500 and 100 mM, respectively. All materials show nearly frequency independent behavior, i.e. G' > G'' from (ω = 0.1 – 100 rad·s^{-1}). Only at high frequency (ω > 10 rad·s^{-1}) the soft materials show a decrease in G', which could be due to either machine limitations (as the raw phase becomes close to

180° at high frequencies) or it might indicate destruction of the materials. Overall, the results show that the **UPy-EG$_{11}$** gel forms a stiffer network (higher G') than the **BTA-EG$_4$** and **Phz-EG$_4$** assemblies.

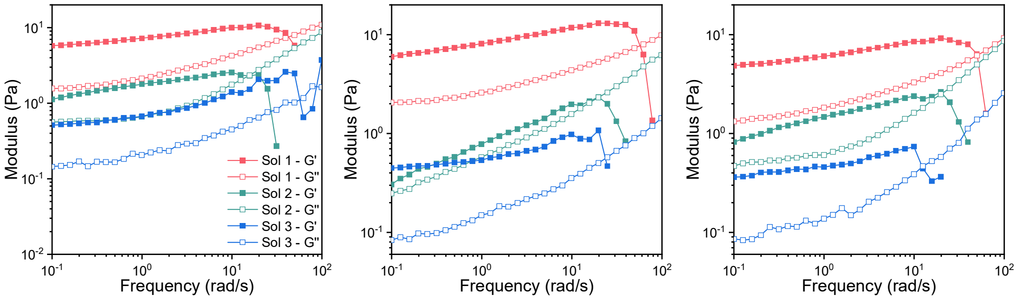

Figure S20: Additional frequency-dependent rheological measurements of solutions phases of the **OTAB**:**UPy-EG$_{11}$**:**Phz-EG$_4$**:**BTA-EG$_4$** system (1:0.024:0.055:0.160) in dilution series of Figure S16 (γ = 1%, from left to right Run 1, 2 and 3). Sol 1, 2 and 3 are at **OTAB** concentrations of 650, 350 and 20 mM, respectively. All materials show nearly frequency independent behavior from ω = 0.1 – 10 rad·s^{-1}, i.e. G' > G''. At higher frequency (ω > 10 rad·s^{-1}) the materials show a decrease in G', which could be due to either machine limitations (as the raw phase becomes close to 180°) or it might indicate destruction of the materials. These phases show significantly lower moduli (G' and G'') than the gel-like phases of the **OTAB**:**UPy-EG$_{11}$**:**Phz-EG$_4$**:**BTA-EG$_4$** system (1:0.024:0.055:0.160) in Figure S16, indicating formation of (near) liquid dispersions instead of gels.

S4. Details on computations with mass-balance models

S4.1 Assembly landscape simulations

S4.1.1 Schematic overview of included aggregation processes

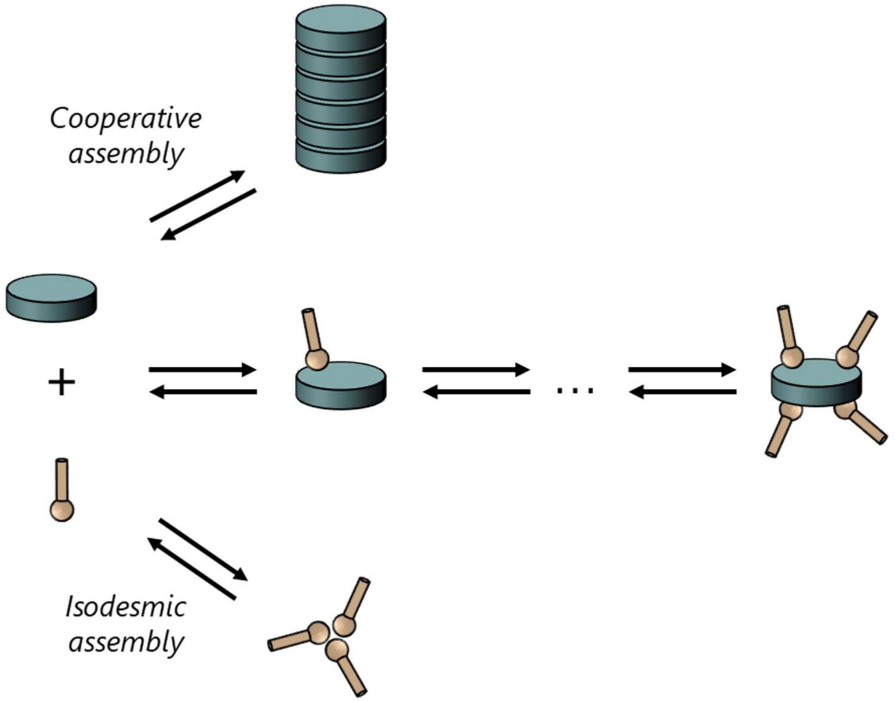

Scheme S1: Schematic representation of aggregation pathways included in the model for **(*S*)-Por$_{\mathbf{Zn}}$** (green disc) with ethanol (beige stick) additive.[S9]

S4.1.2 Details on mass-balance model for assembly with sequestrator additive

The cooperative pathway in supramolecular polymerizations is modelled using thermodynamic mass balance expressions.[S10] In the model, the polymers (H) are assumed to grow through monomer (M) addition and dissociation at the chain ends. The reactions that describe the cooperative pathway, for which a nucleus size of 2 is assumed, are:

$$\begin{aligned} &\mathrm{M}+\mathrm{M} \underset{}{\overset{K_\mathrm{n}}{\rightleftharpoons}} \mathrm{H}_2 \\ &\mathrm{H}_2+\mathrm{M} \overset{K_\mathrm{e}}{\rightleftharpoons} \mathrm{H}_3 \\ &\mathrm{H}_3+\mathrm{M} \overset{K_\mathrm{e}}{\rightleftharpoons} \mathrm{H}_4 \\ &\quad\vdots \\ &\mathrm{H}_j+\mathrm{M} \overset{K_\mathrm{e}}{\rightleftharpoons} \mathrm{H}_{j+1} \\ &\quad\vdots \end{aligned} \tag{S14}$$

with K_n the nucleation equilibrium constant and K_e the elongation equilibrium constant of the nucleated pathway. Assuming the activity of the chemical species is equal to their concentrations, the concentration of monomers in *i*-mer in the cooperative H-aggregates in thermodynamic equilibrium can then be expressed as a function of the free monomer concentration with:

$$[\mathrm{H}_i] = i \cdot \sigma \cdot K_\mathrm{e}^{i-1} \cdot [\mathrm{M}]^i \text{ for } i \geq 2 \tag{S15}$$

where [M] is the equilibrium monomer concentration and σ is the cooperativity parameter, which is $\sigma = K_\mathrm{n}/K_\mathrm{e}$.

The total concentration of M in the system is the sum of concentration of free monomers and of monomers in aggregates:

$$
\begin{aligned}
[\mathrm{M}]_{\mathrm{tot}} &= [\mathrm{H}]_{\mathrm{tot}} + [\mathrm{M}] \\
&= \left(\sum_{i=2}^{\infty} [\mathrm{H}_i] \right) + [\mathrm{M}] \\
&= \left(\sum_{i=1}^{\infty} i \cdot \sigma \cdot K_{\mathrm{e}}^{i-1} [\mathrm{M}]^i \right) - \sigma \cdot [\mathrm{M}] + [\mathrm{M}]
\end{aligned} \tag{S16}
$$

With standard expressions for converging series, the summation in equation (S16) can be solved and the mass-balance equation for the system can be obtained:

$$[\mathrm{M}]_{\mathrm{tot}} = (1-\sigma) \cdot [\mathrm{M}] + \frac{\sigma \cdot [\mathrm{M}]}{(1 - K_{\mathrm{e}} \cdot [\mathrm{M}])^2} \tag{S17}$$

This equation is solved in Python, using a custom written binary search algorithm, to obtain the free monomer concentration. The free monomer concentration is then used to calculate the concentration of nucleated aggregates.

The solvent dependence of K_e to a cosolvent is introduced via:[S11]

$$K_{\mathrm{e}} = \exp\left(\frac{-\Delta G_{\mathrm{coop}}}{R \cdot T} \right) = \exp\left(\frac{-(\Delta G_{\mathrm{coop}}^{\circ} + m_{\mathrm{coop}} \cdot f_{\mathrm{cosolv}})}{R \cdot T} \right) \tag{S18}$$

with R the gas constant, T the temperature, ΔG_{coop}° the Gibbs free energy of elongation of the cooperative polymerization, ΔG_{coop} the cosolvent-corrected Gibbs free energy of elongation of the cooperative polymerization and m_{coop} the solvent dependency parameter of the elongation process to the cosolvent that is present in solvent fraction f_{cosolv}.

The binding constant K_e is rendered temperature-dependent through the van ‘t Hoff expression:

$$K_{\mathrm{e}} = \exp\left(\frac{-\Delta G_{\mathrm{coop}}}{R \cdot T} \right) = \exp\left(\frac{-\Delta H_{\mathrm{coop}} - m_{\mathrm{coop}} \cdot f_{\mathrm{cosolv}}}{R \cdot T} + \frac{\Delta S_{\mathrm{coop}}}{R} \right) \tag{S19}$$

with ΔH_{coop} and ΔS_{coop} the enthalpy and entropy of elongation, respectively.
The nucleation enthalpy, ΔH_n, is introduced via:

$$\Delta H_{\mathrm{n}} = \Delta H_{\mathrm{coop}} + NP \tag{S20}$$

with ΔH_{coop} the enthalpy of elongation and NP a nucleation penalty. The nucleation penalty is related to the cooperativity parameter σ via:

$$\sigma = e^{\frac{-NP}{R \cdot T}} \tag{S21}$$

For the cooperative pathway, the nucleation energy, ΔG_n, is introduced via:

$$\Delta G_{\mathrm{n}} = \Delta G_{\mathrm{coop}} - R \cdot T \ln(\sigma) \tag{S22}$$

which relates to the nucleation constant via:

$$K_{\mathrm{n}} = \exp\left(\frac{-\Delta G_{\mathrm{n}}}{R \cdot T} \right) \tag{S23}$$

The clustering of ethanol in MCH was previously fitted as an isodesmic assembly with equilibrium constant K_{clus}. For isodesmic aggregation pathways, the equations above apply with σ=1, NP=0 and thus K_n=K_e, resulting in the mass-balance equation for this clustering:

$$[\mathrm{S}]_{\mathrm{clus}} = \frac{[\mathrm{S}]}{(1 - K_{\mathrm{clus}}[\mathrm{S}])^2} - [\mathrm{S}] \tag{S24}$$

For the temperature dependence of K_{clus}, we will use the thermodynamic parameters ΔH_{clus} and ΔS_{clus}:

$$K_{\mathrm{clus}} = \exp\left(\frac{-\Delta G_{\mathrm{clus}}}{R \cdot T} \right) = \exp\left(\frac{-\Delta H_{\mathrm{clus}}}{R \cdot T} + \frac{\Delta S_{\mathrm{clus}}}{R} \right) \tag{S25}$$

The interaction between the monomer M (**(*S*)-Por$_{\mathbf{Zn}}$**) and additive S (ethanol) is considered as a sequential addition of S to M up to a 1:k complex MS$_k$:[S12]

$$\begin{aligned} M + S &\xrightleftharpoons{K_{MS}} MS \\ MS + S &\xrightleftharpoons{K_{MS}} MS_2 \\ MS_2 + S &\xrightleftharpoons{K_{MS}} MS_3 \\ &\vdots \\ MS_{k-1} + S &\xrightleftharpoons{K_{MS}} MS_k \end{aligned} \tag{S26}$$

with K_{MS} the binding constant of additive to monomer. It is assumed that k equals the number of amide functional groups in the monomer, which is 4 for **(*S*)-Por$_{Zn}$**.
The concentration of monomers in monomer–additive complexes in thermodynamic equilibrium $[M]_{MS_k}$ can then be expressed as a function of the free monomer concentration with:

$$[M]_{MS_k} = \sum_{i=1}^{k} K_{MS}^{i} \cdot [M] \cdot [S]^i = [M] \cdot \frac{K_{MS} \cdot [S] - (K_{MS} \cdot [S])^{k+1}}{1 - K_{MS} \cdot [S]} \tag{S27}$$

and the concentration of additives in the monomer-additive complex $[S]_{MS_k}$ as:

$$[S]_{MS_k} = \sum_{i=1}^{k} i \cdot K_{MS}^{i} \cdot [M] \cdot [S]^i = [M] \cdot K_{MS} \cdot [S] \cdot \frac{1 - (k+1) \cdot (K_{MS} \cdot [S])^k + k \cdot (K_{MS} \cdot [S])^{k+1}}{(1 - K_{MS} \cdot [S])^2} \tag{S28}$$

Adding these terms to the mass-balance equations yields the mass-balance equations for monomer–additive systems:

$$[M]_{tot} = (1-\sigma) \cdot [M] + \frac{\sigma \cdot [M]}{(1 - K_e \cdot [M])^2} + [M] \cdot \frac{K_{MS} \cdot [S] - (K_{MS} \cdot [S])^{k+1}}{1 - K_{MS} \cdot [S]} \tag{S29}$$

$$[S]_{tot} = \frac{[S]}{(1 - K_{clus} \cdot [S])^2} + [M] \cdot K_{MS} \cdot [S] \cdot \frac{1 - (k+1) \cdot (K_{MS} \cdot [S])^k + k \cdot (K_{MS} \cdot [S])^{k+1}}{(1 - K_{MS} \cdot [S])^2} \tag{S30}$$

The binding constant K_{MS} is rendered temperature-dependent through the van 't Hoff expression:

$$K_{MS} = \exp\left(\frac{-\Delta G_{MS}}{R \cdot T}\right) = \exp\left(\frac{-\Delta H_{MS}}{R \cdot T} + \frac{\Delta S_{MS}}{R}\right) \tag{S31}$$

with ΔH_{MS} and ΔS_{MS} the enthalpy and entropy of the monomer–additive interaction, respectively.

The degree of aggregation was then calculated by:

$$\text{degree of aggregation} = \frac{[H]_{tot}}{[M]_{tot}} \tag{S32}$$

S4.1.3 Thermodynamic parameters for **(*S*)-Por$_{Zn}$** assembly in MCH with EtOH

Table S3: Thermodynamic parameters for **(*S*)-Por$_{Zn}$** with EtOH in MCH as determined in reference S9.

ΔH_{coop} (kJ/mol)	ΔS_{coop} (J/mol/K)	*NP* (kJ/mol)	m_{coop} (kJ/mol)	ΔH_{MS} (kJ/mol)	ΔS_{MS} (J/mol/K)	ΔH_{clus} (kJ/mol)	ΔS_{clus} (J/mol/K)
–70	–97.6	20	2000	–35.6	–89.6	–9.7	–248.0

S4.2 Diluted Majority-rules simulations for covalent copolymer modification

S4.2.1 Details on mass-balance model for three-component copolymerization

To study the supramolecular copolymerization of monomers **A**, **B** and **C** into two competing polymers with opposite helicity (*P* and *M*), the derivation of the mass-balance model was followed, as reported by Ten Eikelder and co-workers.[S13] For each monomer, the Gibbs free energy change upon elongation

in the *M*-polymer ($\Delta G_{M,\mathrm{e}}$) was calculated from the Gibbs free energy change upon elongation in the *P*-polymer ($\Delta G_{P,\mathrm{e}}$) and the mismatch penalty (*MMP)*:

$$\Delta G_{M,\mathrm{e}} = \Delta G_{P,\mathrm{e}} + MMP \tag{S33}$$

which relates to the elongation constant (per example of monomer **A** in *M* polymers) via:

$$K_{M,\mathbf{A\text{-}A}} = \exp\left(\frac{-\Delta G_{M,\mathrm{e}}}{R \cdot T}\right) \tag{S34}$$

The mass-balance equations for the monomers become:

$$[\mathbf{A}]_{\mathrm{tot}} = [\mathbf{A}] + [\mathbf{A}]_P([\mathbf{A}],[\mathbf{B}],[\mathrm{C}]) + [\mathbf{A}]_M([\mathbf{A}],[\mathbf{B}],[\mathrm{C}]) \tag{S35}$$

$$[\mathbf{B}]_{\mathrm{tot}} = [\mathbf{B}] + [\mathbf{B}]_P([\mathbf{A}],[\mathbf{B}],[\mathrm{C}]) + [\mathbf{B}]_M([\mathbf{A}],[\mathbf{B}],[\mathrm{C}]) \tag{S36}$$

$$[\mathbf{C}]_{\mathrm{tot}} = [\mathbf{C}] + [\mathbf{C}]_P([\mathbf{A}],[\mathbf{B}],[\mathrm{C}]) + [\mathbf{C}]_M([\mathbf{A}],[\mathbf{B}],[\mathrm{C}]) \tag{S37}$$

with $[X]_Y$ as the equivalent concentration of monomers *X* in polymer type *Y* if the polymers would fully disassemble and with $[X]_Y([\mathbf{A}],[\mathbf{B}],[\mathbf{C}])$ for Y = *P* or *M*, and X = **A** as the sum of the 4th, 5th and 6th components of the vector $\mathbf{U}_Y$, for X = **B** the 7th, 8th and 9th components and for X = **C** the 10th, 11th and 12th components:

$$\mathbf{U}_Y = (I - N_Y)^{-1} \cdot N_Y \cdot \mathbf{u}_{Y,\mathbf{1}} \tag{S38}$$

with 12x12 identity matrix *I*, and $\mathbf{u}_{Y,\mathbf{1}}$:

$$\mathbf{u}_{Y,\mathbf{1}} = \begin{pmatrix} \sigma_{\mathbf{A}} \cdot [\mathbf{A}] \\ \sigma_{\mathbf{B}} \cdot [\mathbf{B}] \\ \sigma_{\mathbf{C}} \cdot [\mathbf{C}] \\ \sigma_{\mathbf{A}} \cdot [\mathbf{A}] \\ 0 \\ 0 \\ 0 \\ \sigma_{\mathbf{B}} \cdot [\mathbf{B}] \\ 0 \\ 0 \\ 0 \\ \sigma_{\mathbf{C}} \cdot [\mathbf{C}] \end{pmatrix} \tag{S39}$$

and 12x12 matrix N_Y *:*

$$N_Y = \begin{pmatrix} N_{Y,c} & 0 & 0 & 0 \\ N_{Y,\mathbf{A}} & N_{Y,c} & 0 & 0 \\ N_{Y,\mathbf{B}} & 0 & N_{Y,c} & 0 \\ N_{Y,\mathbf{C}} & 0 & 0 & N_{Y,c} \end{pmatrix} \tag{S40}$$

with $N_{Y,c}$*:*

$$N_{Y,c} = \begin{pmatrix} K_{Y,\mathbf{A\text{-}A}} \cdot [\mathbf{A}] & K_{Y,\mathbf{B\text{-}A}} \cdot [\mathbf{A}] & K_{Y,\mathbf{C\text{-}A}} \cdot [\mathbf{A}] \\ K_{Y,\mathbf{A\text{-}B}} \cdot [\mathbf{B}] & K_{Y,\mathbf{B\text{-}B}} \cdot [\mathbf{B}] & K_{Y,\mathbf{C\text{-}B}} \cdot [\mathbf{B}] \\ K_{Y,\mathbf{A\text{-}C}} \cdot [\mathbf{C}] & K_{Y,\mathbf{B\text{-}C}} \cdot [\mathbf{C}] & K_{Y,\mathbf{C\text{-}C}} \cdot [\mathbf{C}] \end{pmatrix} \tag{S41}$$

and $N_{Y,X}$:

$$N_{Y,X} = \begin{pmatrix} K_{Y,\mathbf{A\text{-}A}} \cdot [\mathbf{A}] \cdot \delta_{X\mathbf{A}} & K_{Y,\mathbf{B\text{-}A}} \cdot [\mathbf{A}] \cdot \delta_{X\mathbf{A}} & K_{Y,\mathbf{C\text{-}A}} \cdot [\mathbf{A}] \cdot \delta_{X\mathbf{A}} \\ K_{Y,\mathbf{A\text{-}B}} \cdot [\mathbf{B}] \cdot \delta_{X\mathbf{B}} & K_{Y,\mathbf{B\text{-}B}} \cdot [\mathbf{B}] \cdot \delta_{X\mathbf{B}} & K_{Y,\mathbf{C\text{-}B}} \cdot [\mathbf{B}] \cdot \delta_{X\mathbf{B}} \\ K_{Y,\mathbf{A\text{-}C}} \cdot [\mathbf{C}] \cdot \delta_{X\mathbf{C}} & K_{Y,\mathbf{B\text{-}C}} \cdot [\mathbf{C}] \cdot \delta_{X\mathbf{C}} & K_{Y,\mathbf{C\text{-}C}} \cdot [\mathbf{C}] \cdot \delta_{X\mathbf{C}} \end{pmatrix} \tag{S42}$$

where X = **A**, **B** or **C** and: $\delta_{ij} = \begin{cases} 0 \text{ if } i \neq j, \\ 1 \text{ if } i = j. \end{cases}$

The mass-balance equations are solved in the model with a nested binary search algorithm.

It was assumed that the molar ellipticity of the free monomers is 0. For the copolymerization, the simulated CD signal was then calculated by:

$$\mathrm{CD_{calc}} = \frac{\theta_{\mathbf{A},p} \cdot ([\mathbf{A}]_P - [\mathbf{A}]_M) + \theta_{\mathbf{B},p} \cdot ([\mathbf{B}]_P - [\mathbf{B}]_M) + \theta_{\mathbf{C},p} \cdot ([\mathbf{C}]_P - [\mathbf{C}]_M)}{[\mathbf{A}]_{\mathrm{tot}} + [\mathbf{B}]_{\mathrm{tot}} + [\mathbf{C}]_{\mathrm{tot}}} \tag{S43}$$

with $\theta_{X,p}$ being the molar ellipticity of monomer X in P-polymers and having the opposite sign for monomer X in M-polymers.

S4.2.2 Thermodynamic parameters for BTA copolymerization in MCH

Table S4: Thermodynamic parameters for assembly of **a-BTA** (A), **Glu(OMe)-BTA** (B) and **Glu-BTA** (C) in MCH, approximated by manually fitting the model of section S4.2.1 to the data in reference S5.

**a-BTA**	ΔH_A (kJ/mol)	ΔS_A (J/mol/K)	NP_A (kJ/mol)	MMP_A (kJ/mol)
	–90.0	–139.8	8.9	0
**Glu(OMe)-BTA**	ΔH_B (kJ/mol)	ΔS_B (J/mol/K)	NP_B (kJ/mol)	MMP_B (kJ/mol)
	–77.0	–140.0	10.0	4
**Glu-BTA**	ΔH_C (kJ/mol)	ΔS_C (J/mol/K)	NP_C (kJ/mol)	MMP_C (kJ/mol)
	–67.0	–140.0	10.0	1

S5. Python scripts used for Bayesian optimization

The following Python scripts (see Code Availability) were used to perform and validate Bayesian optimization of supramolecular systems:

- BO_AssemblyLandscapes.py (Results in Figure 2b)
  for Bayesian optimization to map assembly landscape of porphyrin monomers with ethanol additives in methylcyclohexane, using data simulated with mass-balance models.

- BO_AssemblyLandscapes_BatchSize_and_NoisyMeasurements.py (Results in Figure 2d and e)
  for Bayesian optimization to map assembly landscape of porphyrin monomers with ethanol additives in methylcyclohexane, using data simulated with mass-balance models. This script can be used to run BO with larger experimental batch sizes and artificial noise on simulated data.

- BO_AssemblyLandscapes_ExperimentalValidation.py (Results in Figure 2f)
  for experimental Bayesian optimization to map assembly landscape of porphyrin monomers with ethanol additives in methylcyclohexane.

- AssemblyLandscape_Simulator.py
  for simulating data used in Bayesian optimization of BO_AssemblyLandscapes scripts, using the mass-balance model for porphyrin monomers with ethanol additives in methylcyclohexane.

- AssemblyLandscape_Solver.py
  for solving the mass-balance equations for porphyrin monomers with ethanol additives in methylcyclohexane.

- BO_CovalentModification.py (Results in Figure 3c-e)
  for Bayesian optimization to map landscape of change in CD upon covalent modification of chiral BTA in three-component copolymers, using data simulated with mass-balance models.

- BO_CovalentModification_Simulator.py
  for simulating data used in Bayesian optimization of BO_CovalentModification script, using the mass-balance model for three-component BTA copolymers in methylcyclohexane.

- BO_CovalentModification_Solver.py
  for solving the mass-balance equations for three-component BTA copolymers in methylcyclohexane.

- BO_Monomer_Surfactant.py (Results in Figure 4c, d and f)
  for experimental Bayesian optimization to map phase diagrams of water-soluble monomers with OTAB as surfactant additive in water.

S6. References

S1. Dai, C. & Glotzer, S. C. Efficient Phase Diagram Sampling by Active Learning. *J. Phys. Chem. B* **124**, 1275–1284 (2020).

S2. Jones, D.R., Schonlau, M., Welch, W.J. Efficient Global Optimization of Expensive Black-Box Functions. *J. Glob. Optim.* **13**, 455–492 (1998).

S3. Helmich, F. et al. Dilution-induced self-assembly of porphyrin aggregates: A consequence of coupled equilibria. *Angew. Chem. Int. Ed.* **49**, 3939–3942 (2010).

S4. Roosma, J., Mes, T., Leclère, P., Palmans, A. R. A. & Meijer, E. W. Supramolecular Materials from Benzene-1,3,5-Tricarboxamide-Based Nanorods. *J. Am. Chem. Soc.* **130**, 1120–1121 (2008).

S5. de Graaf, F. V., Jansen, S. A. H., Schnitzer, T., Meijer, E. W. & Vantomme, G. Controlling Helical Asymmetry in Supramolecular Copolymers by In Situ Chemical Modification. *J. Am. Chem. Soc.* **145**, 14379–14386 (2023).

S6. Kieltyka, R. E. et al. Mesoscale modulation of supramolecular ureidopyrimidinone-based poly(ethylene glycol) transient networks in water. *J. Am. Chem. Soc.* **135**, 11159–11164 (2013).

S7. de Graaf, F.V. et al. Supramolecular polymerization of hydrogen-bonded trimers in bulk and aqueous medium. *CCS Chem.* **6**, 1468–1465 (2024).

S8. Leenders, C. M. A. et al. Supramolecular polymerization in water harnessing both hydrophobic effects and hydrogen bond formation. *Chem. Commun.* **49**, 1963–1965 (2013).

S9. Jansen, S. A. H. et al. Simulating Assembly Landscapes for Comprehensive Understanding of Supramolecular Polymer-Solvent Systems. *J. Am. Chem. Soc.* **145**, 4231–4237 (2023).

S10. Zhao, D. & Moore, J. S. Nucleation-elongation: A mechanism for cooperative supramolecular polymerization. *Org. Biomol. Chem.* **1**, 3471–3491 (2003).

S11. Mabesoone, M. F. J. et al. Competing Interactions in Hierarchical Porphyrin Self-Assembly Introduce Robustness in Pathway Complexity. *J. Am. Chem. Soc.* **140**, 7810–7819 (2018).

S12. Rao, K. V. et al. Distinct Pathways in 'Thermally Bisignate Supramolecular Polymerization': Spectroscopic and Computational Studies. *J. Am. Chem. Soc.* **142**, 598–605 (2020).

S13. Ten Eikelder, H. M. M., Adelizzi, B., Palmans, A. R. A. & Markvoort, A. J. Equilibrium Model for Supramolecular Copolymerizations. *J. Phys. Chem. B* **123**, 6627–6642 (2019).
